# Targeting the DNA damage response prevents regrowth of colorectal peritoneal metastasis-derived organoids following treatment with mitomycin C

**DOI:** 10.1038/s41416-025-03310-z

**Published:** 2026-01-05

**Authors:** Kyah van Megesen, Arianna Stefan, Madelief Kieboom, Rebecca Amico, Anne M. L. Jansen, Inne Borel Rinkes, Onno Kranenburg

**Affiliations:** 1 Department of Surgical Oncology, University Medical Center Utrecht, Utrecht, The Netherlands; 2 Department of Pharmacy, School of Medicine and Surgery, University of Naples Federico II, Naples, Italy; 3 Department of Pathology, University Medical Center Utrecht, Utrecht, The Netherlands; 4 Utrecht Platform for Organoid Technology, Utrecht University, Utrecht, The Netherlands

**Keywords:** Cancer therapeutic resistance, Colorectal cancer

## Abstract

**Background:**

A minority of Colorectal Cancer (CRC) patients with peritoneal metastases is eligible for cytoreductive surgery (CRS) followed by hyperthermic intraperitoneal chemotherapy (HIPEC). However, recurrence rates are high (~80%). We tested whether inhibitors of the DNA damage response (DDR) could prevent recurrence in an in vitro HIPEC model.

**Methods:**

Peritoneal metastasis-derived organoids (PMDOs; *n* = 10) were treated with inhibitors of ATR (berzosertib, ceralasertib, elimusertib), CHK1 (rabusertib), and WEE1 (adavosertib) alone, and in combination with MMC, oxaliplatin, or irinotecan. Western blotting was used to determine Chk1 phosphorylation and markers of DNA damage. Microscopy and ATP quantification were used to measure the effects of (combination) treatments on cell viability and recurrence/regrowth.

**Results:**

All PMDOs displayed rapid regrowth (recurrence) following single-drug chemotherapy treatment. Berzosertib inhibited chemotherapy-induced CHK1 phosphorylation, augmented DNA damage, and abrogated recurrence in all 10 MMC-treated PMDOs. The combination with oxaliplatin and irinotecan was less effective. In vitro HIPEC with MMC, followed by ‘adjuvant’ treatment with any of the DDR inhibitors for 3 days, completely prevented PMDO recurrence.

**Conclusions:**

PMDOs can be completely eradicated if MMC treatment is followed by inhibition of ATR or other DDR kinases. DDR inhibitors may therefore have value in the adjuvant treatment of peritoneal metastases following CRS-HIPEC.

## Background

The presence of peritoneal metastases (PM) in patients with colorectal cancer (CRC) is associated with an extremely poor prognosis [1]. Once PM is diagnosed, the only treatment that results in long-term survival involves radical surgery to remove all visible disease (cytoreductive surgery; CRS). The addition of hyperthermic intra-peritoneal chemotherapy (HIPEC) may improve survival by killing any remaining micro-metastases [2, 3]. However, approximately half of the patients experience rapid disease progression within the first year after CRS-HIPEC, which further increases to 70% after 2 years [4]. Eventually, 10-year overall survival following CRS-HIPEC is only ~20% [4–7]. Clearly, more efficient treatment strategies are urgently needed to prevent recurrence following CRS/HIPEC.

The drug most commonly used in HIPEC is Mitomycin C (MMC). In addition, oxaliplatin and irinotecan are used for the intraperitoneal treatment of inoperable PM in clinical trials [8–11]. MMC is an alkylating agent that causes inter-strand cross-links. Upon such damage, DNA damage response (DDR) mechanisms are activated. These repair processes temporarily create single-stranded DNA (ssDNA) regions as intermediate steps in DNA repair [12]. ssDNA attracts Replication Protein A (RPA), which recruits ataxia telangiectasia and Rad3-related protein kinase (ATR). ATR phosphorylates Checkpoint kinase 1 (CHK1) which results in activation of both the S- and M-phase cell cycle checkpoints, thus allowing DNA repair before mitosis [13, 14]. Moreover, all checkpoints are governed by cyclin-dependent kinases (CDKs), and activation of CDK1 (Cdc2) is needed for progression to mitosis. Wee1 kinase causes inhibitory phosphorylation of CDK1, thereby inactivating CDK1-cyclin B thus keeping cells in G2 [15, 16].

Targeting the DNA damage response is a promising strategy to limit recurrence following chemotherapy exposure, and can be achieved by combining DNA-damaging agents with DDR inhibitors [14, 17, 18]. Theoretically, DDR inhibition would cause cell-cycle progression and entry into mitosis in the presence of DNA replication stress and unrepaired DNA damage, thereby triggering mitotic catastrophe and apoptosis [19]. In recent years, the research field on DDR inhibitors as anti-cancer therapeutics has expanded tremendously [20]. Pharmaceutical companies are now testing various DDR inhibitors in clinical trials, alone or in combination-treatment strategies.

Multiple inhibitors of the DDR are currently being tested in the clinic [20]. Nevertheless, clinical trials often fail at least in part because the pre-clinical models lack the complexity and treatment history of late-stage cancer patients who participate in trials [21, 22]. To address this and maximize the chance of generating relevant results, we used organoids derived from the relevant disease entity in the current study (i.e., PM). Organoid technology is currently considered one of the most relevant culturing platforms for colorectal cancer and other types of cancer [23, 24]. Indeed, treatment responses measured in organoids appear to reflect the responses observed in cancer patients [25, 26]. Moreover, organoid biobanks are widely being used to devise novel (combination) treatment strategies.

We previously showed that ATR inhibition increases the efficacy of MMC treatment of PM-derived organoids (PMDOs) from CRC in an in vitro HIPEC model [27]. Here, we applied the HIPEC model to study recurrence by tracking long-term regrowth potential following exposure of PMDOs to MMC, oxaliplatin, and irinotecan. To this end, we used a heterogeneous population of 10 PMDOs in which the major histological PM subtypes are represented (i.e., adenocarcinoma (AC), mucinous carcinoma (MC), and signet ring cell carcinoma (SRC)). We modeled recurrence by growing cells for up to 8 weeks, which allows for surviving cells to grow out into full organoids after treatment. These PMDOs were used to assess whether pharmaceutical targeting of key players in the DDR (ATR, CHK1, Wee1) could prevent long-term regrowth (i.e., recurrence) following treatment with MMC, oxaliplatin, or irinotecan. We also studied the effect of treatment duration and application of heat.

The results demonstrate that DDR targeting is a highly effective strategy to prevent PMDO recurrence following short-term treatment with MMC, regardless of heat application or histological subtype. The possibilities for clinical translation of the results are discussed in the context of CRS-HIPEC.

## Methods

### PM-derived organoids

The current study used ten different PMDOs from ten PM-CRC patients. All PMDOs are part of a large living biobank (manuscript in preparation). Some have been described before (Table S1) [27, 28]. Inclusion criteria of PIPAC-I (NCT03246321) [29, 30], PIPAC-II (NL8303) [31], and Interact-II (NCT06003998) [32] applied, or patients were included through an unpublished collaboration (ORCAPM) between UMC Utrecht and Catharina Hospital Eindhoven (Table S1). For the latter, all adult patients with metastases of gastrointestinal tract tumors into the abdominal cavity, undergoing any procedure in which ascites, tumor tissue (and possibly normal surrounding tissue) becomes available as a direct result of the treatment plan could be included. For this study, only PM-CRC material, including ascites, was used. All patients gave their written informed consent for PMDO generation after a consideration period. Organoid and matching patient details are described in Table S1.

### Organoid culture

PMDOs were dissociated into single cells using TrypLE™ (Gibco, 12604021) and plated out using BME matrix (Cultrex Reduced Growth Factor Basement Membrane Extract, Type 2, Pathclear; R&D Chemicals). After solidification for 30 min at 37 °C, full CRC organoid growth medium (BM2 + + or BM3 + ) was added and refreshed twice a week. Full CRC organoid growth medium consisted of basal medium supplemented with niche factors. The basal medium used was Advanced DMEM/F12 (Gibco, Life Technologies Corporation, 12634-010, Grand Island, NY, USA), supplemented with 400 µM Glutamax (Life Technologies, 35050038), 10 mM HEPES (Lonza, BE17-737E, Basel, Switzerland), and 50 U/mL–50 µg/mL penicillin–streptomycin (5.000 U/mL & 5.000 µg/mL; Life Technologies Corporation, 15070063). The niche factors used for BM2 + + were 100 ng/mL noggin conditioned medium, 1× B27 (Life Technologies, 17504044), 500 nM A83-01 (Biovision, 1725-1, Zurich, Switzerland), 10 µM SB202190 (Sigma-Aldrich, S7067, St Louis, MO, USA), 1,25 mM N-Acetyl-L-cysteine (NAC, Sigma-Aldrich, A9165), 10 mM Nicotinamide (Sigma-Aldrich, N0636-100gr), ng/mL EGF (Sigma-Aldrich, E9644), 10 nM Gastrin [Leu15] (Sigma-Aldrich, G9145), and 10 nM prostaglandin E2 (Tocris, 2296-10, Bio-Techne Ltd., Abingdon, UK). The niche factors used for BM3+ were 100 ng/mL noggin conditioned medium, R-Spondin conditioned medium, 1× B27 (Life Technologies, 17504044), 500 nM A83-01 (Biovision, 1725-1, Zurich, Switzerland), 1,25 mM N-Acetyl-L-cysteine (Sigma-Aldrich, A9165), ng/mL EGF (Sigma-Aldrich, E9644), 10 nM Gastrin [Leu15] (Sigma-Aldrich, G9145), 100 ng/ml IGF (BioLegend, 590904), 50 ng/ml FGF (PeproTech, 100-18B), and 10 µM Rock inhibitor (Sigma Aldrich, Y-27632). All PMDOs were grown in BM2 + + except for PMDO054D, PMDO059, and PMDO20-1, which were grown in BM3 + . Organoid morphology is shown in Fig. S1A. Organoids were tested for mycoplasma contamination every two months.

### Organoid DNA isolation and sequencing

Tumor organoids were grown for three days, harvested using TrypLE, and collected in PBS. Total DNA was isolated using the DNeasy Mini Kit (Qiagen) according to the manufacturer’s instructions. The concentration and quality of the extracted genomic material were measured before sequencing.

For PMDO02-1, 03-1, 12-1, and 20-1, whole-genome sequencing (WGS) was performed on an Illumina NovaSeq 6000 (2x150bp). Mapping, single-nucleotide variant (SNV) and InDel detection, copy-number, and structural-variation calling were performed using the “Illumina Analysis Pipeline” of the Utrecht Bioinformatics Expertise Core within the Center for Molecular Medicine at the UMCU, as described before [33]. Sequencing of other organoids was performed by GenomeScan Leiden using the Illumina TruSight Oncology 500 assay and analyzed through R2 (https://hgserver1.amc.nl).

All variants detected in the following gene list were classified according to ACMG guidelines into five classes, from benign (class 1) to pathogenic (class 5) [34]. Only variants classified as class 4 (likely pathogenic) or class 5 (pathogenic) are reported. The following list of genes was assessed for all PMDOs: AKT1, AMER1, APC, AR, ARID1A, ATM, ATR, BARD1, BCOR, BRAF, BRCA1, BRCA2, BRIP1, CALR, CDK12, CHEK1, CHEK2, CCND3, EGFR, FANCA, FANCL, FBXW7, FGFR1, KRAS, MAP2K1, MAP2K4, MAP3K1, MDM2, MLH1, MSH2, MSH3, MSH6, MTOR, NTRK1, NRAS, PALB2, PDGFRB, PIK3CA, PMS2, PPP2R2A, PRKAR1A, PRKCI, RAD51B, RAD51C, RAD51D, RAD54L, RB1, RICTOR, RNF43, RUNX1, SMAD2, SMAD4, SMARCA4, SMARCB1, TCFL2, TCF7L2, TP53, and ZFHX3. Mutation data are presented in Table S2.

### Western blot analysis

PMDOs were dissociated into single cells using TrypLE™, plated out in 50% BME matrix, and grown for 3 days in full CRC organoid medium. The medium was changed to basal medium, and compounds were added in the indicated concentrations. PMDOs were harvested at the indicated time points using dispase, washed with PBS, and lysed in Laemmli lysis buffer (2.5% SDS, 20% glycerol, 120 mM TRIS pH 6.8). Equal amounts of protein (10–20 μg), as determined by the Lowry method [35], were run on SDS-PAA gels with PageRuler™ Plus Prestained Protein Ladder (26620, Thermo Fisher Scientific, Breda, The Netherlands). Gels were transferred onto nitrocellulose membranes (Trans-Blot Turbo, Bio-Rad, Hercules, CA, USA) using a Trans-blot Turbo transfer system (1704158, BioRad, Lunteren, The Netherlands). Unspecific binding was prevented by blocking for 1 h with 5% milk in PBS, after which membranes were incubated with primary antibodies overnight at 4 °C. Primary antibodies used were pChk1 (Ser345, Cell Signaling, 2348), Chk1 (Cell Signaling, 2360), yH2Ax (Ser139, Millipore, 05-636), cleaved PARP (Cell Signaling, 9541), pRPA32 (S4/S8, Bethyl, A300-245A), and β-actin (Novus, NB600-501). Next, membranes were washed three times with 1× TBS for 5 min at RT and incubated with HRP-conjugated secondary antibodies (DAKO, P0447 & P0448) for 1 h at RT. Membranes were washed again, and protein detection was performed using enhanced chemiluminescence (ECL) reagents (Amersham ECL, GE Healthcare, RPN2235/2209, Chicago, IL, USA). To determine relative protein quantities, blot images were analyzed using FUIJI (version1). Using the Gel analyzing tool, the area under the peaks was determined and exported to Excel.

### Fluorescence microscopy

PMDOs were grown, treated, and harvested as for western blotting. Subsequently, cells were fixed with 4% paraformaldehyde for 10 min at RT, permeabilized with 0.2% Triton X-100 in dPBS for 5 min at RT, washed, and incubated with 2% Bovine Serum Fraction V albumin (BSA) in PBS for 30 min at RT to block non-specific binding. Fixed cells were incubated with the primary antibody yH2Ax (1:100, Ser139, Millipore, 05-636) in 2% BSA-PBS at 4 °C overnight, washed 3 times with dPBS, incubated with secondary antibody Goat anti-Mouse IgG-Alexa Fluor 488 (1:500, Invitrogen, A11029) in 2% BSA-PBS for 1 h at RT, washed with dPBS, then with demi water, and finally with 70% EtOH. The pellet was resuspended in EtOH and pipetted onto the glass slide. ProLong™ Gold Antifade Mountant with DNA Stain DAPI (P36934, Invitrogen) was used to mount the cover glass. Images were taken at Zeiss LSM 700 confocal microscope (Carl Zeiss, Germany). To quantify the fluorescent signal, FUIJI (version1) was used. Using threshold, the area of DAPI or yH2AX signal was measured and exported to Excel.

### Apoptosis assessment

PMDOs were dissociated into single cells using TrypLE™, plated in a 50% BME matrix, and grown for 3 days in full CRC organoid medium. The medium was replaced with fresh culture medium, and compounds were added in the indicated concentrations. To detect activated caspase-3/7 in live cells, 3-days after treatment, CellEvent™ Caspase-3/7 (1:1000, C10423, Thermo Fisher Scientific, Carlsbad, CA, USA) was added to the culture medium. 30 min later, cells were imaged at the Zeiss Cell Observer (Carl Zeiss, Germany). To quantify the fluorescent signal, FUIJI (version1) was used. Using find maxima, Caspase-3/7 signal foci were counted, and results were exported to Excel.

Apoptosis after short-term regrowth was assessed using the Dead Cell Apoptosis Kit with Annexin V-FITC and Propidium Iodide (cat. V13242, Thermo Fisher Scientific, Waltham, MA, USA) according to the manufacturer’s instructions. Organoids were plated and treated as above for 4 days. Following treatment, wash-out, and regrowth, organoids were dissociated into single cells, collected, and washed twice with PBS. The cells were then stained with Annexin V-FITC and Propidium Iodide (PI). Flow cytometry analysis was performed using a BD FACSCelesta™ flow cytometer (BD Biosciences, San Jose, CA, USA). A minimum of 20,000 events per sample were collected. Data were analyzed using FlowJo v10 software (Tree Star, Ashland, OR, USA).

### In vitro drug screen, HIPEC, and PMDO regrowth assessment

Drug response evaluation was performed on three-day-old organoids. 96-well assays were used for direct and paired regrowth screens for both three-day drug incubation screens and in vitro HIPEC. PMDOs were dissociated into single cells using TrypLE™, washed in PBS, counted, and resuspended at a density of 3000 single cells per well in basal growth medium, and then plated in a 75% BME matrix. This was done to preserve droplet integrity over time. There is no effect of BME concentration on morphology and cell growth or drug sensitivity (Fig. S1B-D). Full CRC organoid growth medium was added after solidification for 20 minutes at 37 °C, and the cells were allowed to grow for three days. Then, the medium was replaced with appropriate culture medium, and Mitomycin C (MMC, Selleckchem, S8146), oxaliplatin (Fresenius Kabi), irinotecan (Fresenius Kabi), berzosertib (TargetMol, T2669), camonsertib (MedChemExpress, HY-139609), ceralasertib (TargetMol, T3338), elimusertib (TargetMol, T7318), rabusertib (Selleckchem, LY2603618), or adavosertib (TargetMol, T2077) were added. The exact concentrations and combinations tested are shown in the drug screen layouts in the supplementary figures corresponding to the drug-screen results.

For screens with oxaliplatin, NAC was not used in the culture medium as it detoxifies the drug [28]. Drugs diluted in dimethylsulphoxide (DMSO) or 0.3% Tween-20 were dispensed using the Tecan D300e Digital Dispenser (Tecan Trading AG, Switzerland). Brightfield images of wells were taken with an inverted microscope (EVOS) or the CellInsight^TM^ CX5 High Content Screening Platform (Thermo Fisher Scientific, United States).

For three-day drug incubation screens, cells were incubated with the drugs for 3 days after which the direct plates were imaged, and cell viability was measured. For regrowth plates, droplets were washed three times with PBS, and fresh culture medium was added. The culture medium was refreshed twice a week, and the plates were imaged weekly. At the end of the experiment, plates were imaged, and cell viability was measured.

For in vitro HIPEC or NIPEC (normothermic intraperitoneal chemotherapy), MMC was added to three-day-old organoids as above, and plates were incubated for 90 min at 42 °C or 37 °C, respectively. Droplets were washed three times with PBS, and fresh culture medium was added. After ~20 h, DDR inhibitors were added, and the plate was incubated for three days at 37 °C. Direct plates were imaged, and cell viability was measured. For regrowth plates, droplets were washed three times with PBS, and fresh culture medium was added. Culture medium was refreshed two times per week, and plates were imaged weekly. At the end of the experiment, plates were imaged, and cell viability was measured. Experiment duration for regrowth varied per experiment and is noted in the supplementary figures corresponding to the drug-screen results.

### Cell viability assay following drug screens and regrowth

Cell viability was measured using CellTiter-Glo® 3D Cell Viability Assay (Promega Corporation, G9681, Maddison, WI, USA) according to the manufacturer’s instructions, and luminescence was measured using the SpectraMax® M Series Multi-Mode Microplate Reader (Molecular Devices, LLC). In vitro drug screen and cell viability assay were performed in triplicate or quadruplicate as described above.

### Data analysis and statistics

Image analysis data were loaded into GraphPad Prism version 9.0.0 for Windows (GraphPad Software, San Diego, California, USA, www.graphpad.com). One-way ANOVA was performed, followed by a Tukey’s test for multiple comparisons.

The effects of Berzosertib, Chemotherapy, and their interaction on cell viability were analyzed using a repeated measures ANOVA. The plotted cell viability data corresponds to the concentrations listed in Tables S3-6.

DDR inhibitor (Present, Absent) and Chemotherapy (None, MMC, Oxaliplatin, Irinotecan) were included as fixed effects, with PMDO as a random effect to account for repeated measures within experiments. Sphericity was assessed using Mauchly’s test, and where violated, p-values were adjusted using the Greenhouse-Geisser correction. Significance was set at *p* < 0.05.

Post-hoc analyses were conducted using estimated marginal means (EMMs) to explore significant main effects and interactions:

Tukey’s Test: Pairwise comparisons of the levels of the DDR inhibitor were performed within each level of Chemotherapy to identify treatment differences.

Dunnett’s Test: Within each level of DDR inhibitor (Present, Absent), levels of Chemotherapy were compared against the control (None).

P-values were adjusted separately for each post-hoc testing framework to account for multiple comparisons without overcorrection. Statistical analyses were performed in R (v4.4.0) using the afex, emmeans, and multcomp packages.

Synergy assessment was based on the Additive model [36, 37]. As a measure, the Combination Index (CI) was calculated using the measured cell viability for the combination therapy divided by the predicted cell viability. Cell viability was predicted using the following formula: cell viability monotherapy 1 (%) x cell viability of monotherapy 2 (%) x 0.01. The predicted cell viability may result in false CI scores if the prediction is very low. Thus, a minimum of 5% was maintained for the predicted cell viability. CI > 1.2 was considered a sub-additive (SA) effect, CI 0.8-1.2 implies an additive (Add) effect, CI < 0.8 was classified to be a synergistic (S) effect, and CI < 0.5 indicates a strong synergistic (SS) effect.

Pearson correlation coefficients were computed to assess the association between binary mutation status (class 4 and 5 variants vs no mutation) or pretreatment status and continuous CI values across cell lines. Multiple testing correction was applied using the Benjamini-Hochberg (BH) method to control for false discovery rate (FDR) across gene-wise comparisons. Statistical analyses were performed in R (v4.4.0), and significance was defined as adjusted *p* < 0.05.

## Results

### Berzosertib prevents chemotherapy-induced CHK1 phosphorylation in PMDOs and sensitizes them to chemotherapy

**Fig. 1:**
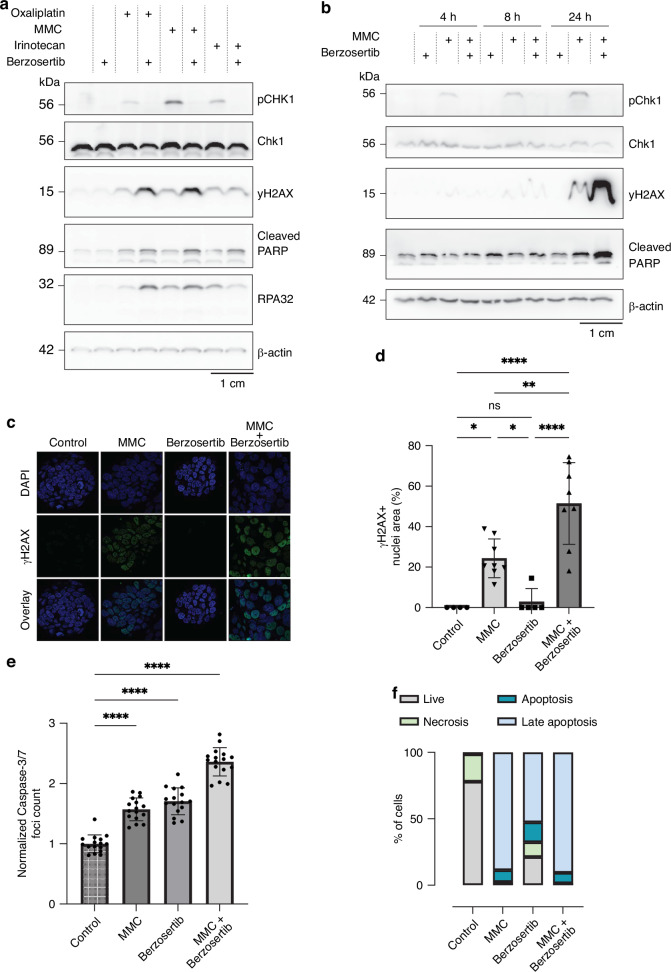
Berzoserib inhibits chemotherapy-induced CHK1 phosphorylation and acts as a chemosensitizer by promoting DNA damage and apoptosis. **a** Western blot analysis of pChk1 (Ser345), Chk1, yH2Ax (Ser139), cleaved PARP, pRPA32 (S4/S8), and β-actin protein expression levels in PMDO14-2 organoids following exposure to 10 μM MMC, 220 μM oxaliplatin, or 128 μM irinotecan, and 5 μM berzosertib or the combination for 24 h. **b** Western blot analysis of pChk1, Chk1, yH2Ax, cleaved PARP, and β-actin protein expression levels in PMDO14-2 organoids following exposure to 10 μM MMC, 220 μM oxaliplatin, or 128 μM irinotecan, and 5 μM berzosertib or the combination for the indicated durations. (24 h samples same as Fig. 1a) **c** Immunofluorescence yH2Ax staining in nuclei of PMDOs (PMDO12-1) treated with 1 μM Berzosertib, 3 μM MMC, or the combination for 24 h. **d** Quantification of nuclei staining of yH2AX as a percentage of total nuclei area. * *P* < 0.05, ** *P* < 0.01, **** *P* < 0.0001, one-way ANOVA with Tukey multiple comparisons. **e** Quantification of fluorescence Caspase-3/7 signal after 3-day treatment of PMDO14-2 with 10 μM MMC, 5 μM berzosertib, or the combination. **** *P* < 0.0001, one-way ANOVA with Tukey multiple comparisons. **f** Stacked bar plot showing the distribution of live, necrotic, apoptotic, and late apoptotic cells, quantified by Annexin V/PI staining and flow cytometry. PMDOs (PMDO12-1) were treated for four days with 10 μM MMC, 5 μM berzosertib, or the combination, followed by a 3-day recovery period.

PMDOs were treated for 24 h with MMC, Irinotecan, Oxaliplatin, or the ATR inhibitor berzosertib [38] alone, or the combination of chemotherapy and berzosertib. Western blot analysis of PMDO lysates showed that all chemotherapeutics induced CHK1 phosphorylation on Ser345 (Fig. 1a). The addition of berzosertib completely inhibited CHK1 phosphorylation in 3 independent PMDOs treated with all three drugs (Fig. 1a and Fig. S2A-E). Berzosertib treatment augmented MMC- and oxaliplatin-induced DNA damage (yH2AX), replication stress (phospho-RPA), and apoptosis (cleaved PARP), but this was not observed with irinotecan.

Furthermore, we followed treatment-induced activation of ATR (phospho-CHK1), DNA damage (yH2AX), and apoptosis (cleaved PARP) over time (4 h, 8 h, and 24 h) using western blotting. MMC treatment caused progressive activation of ATR over time, and this was completely inhibited by co-treatment with berzosertib (Figs. 1b, S2F). When compared to the single drug treatments, the combination treatment resulted in a strong increase in yH2AX and cleaved PARP after 24 h.

To further evaluate DNA damage induction in PMDOs, we performed immunofluorescence analysis of γH2AX, a well-established marker for double-strand DNA breaks Untreated and berzosertib-treated PMDOs showed no measurable yH2AX foci (Fig. 1c, d). MMC induced yH2AX foci to some extent, but this was significantly increased following MMC-berzosertib combination treatment (Fig. 1c, d).

Additionally, apoptosis was assessed immediately after treatment using Caspase-3/7 fluorescence imaging and following short-term regrowth using Annexin-V staining and flow cytometry. Following a three-day treatment with MMC, berzosertib, or their combination, Caspase-3/7 activity was significantly increased (Figs. 1e, S2G-I). After a 4-day treatment with MMC, berzosertib, or MMC and berzosertib, followed by a 3-day regrowth period, both apoptosis and late apoptosis were increased (Figs. 1f, S2J).

Together, these findings demonstrate that the combination of MMC and berzosertib induces pronounced DNA damage and apoptosis in PMDOs, supporting that ATR inhibition enhances chemotherapeutic response.

### Berzosertib prevents PMDO regrowth following MMC treatment

**Fig. 2:**
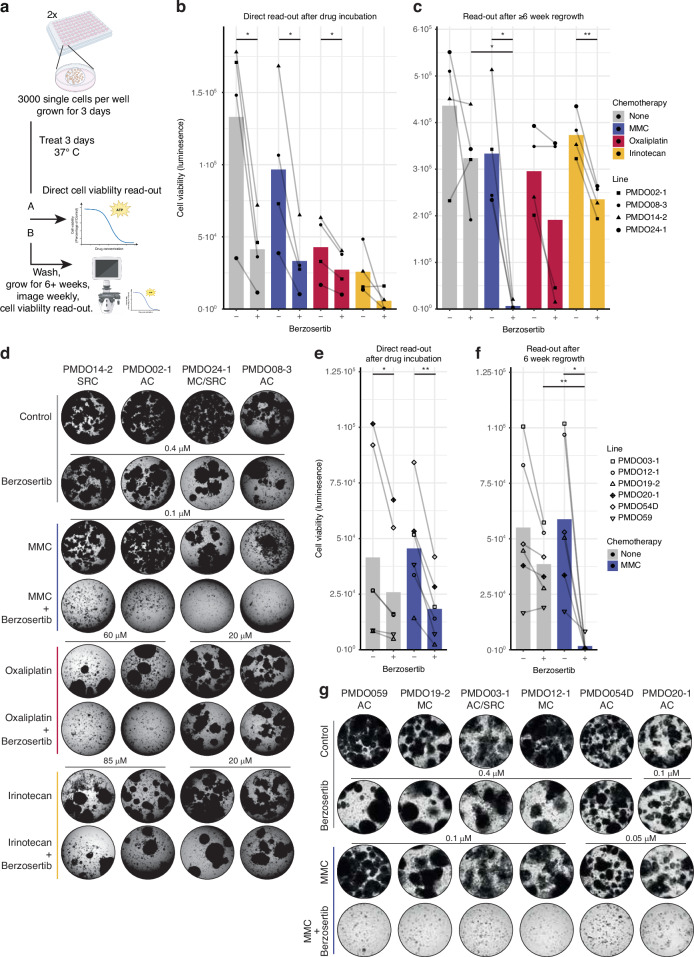
Three-day MMC-berzosertib combination treatment prevents PM-CRC organoid regrowth. **a** Experimental setup of the regrowth experiments. **b** Four distinct PMDOs were treated with MMC(0.1 µM), oxaliplatin (20–60 µM), or Irinotecan (20–85 µM), and berzosertib (0.4 µM) alone and in combination for 72 h. Cell Titer Glo measurements were used to determine cell viability directly after treatment. Graphs show raw luminescence data. Each dot represents *n* ≥  3 per condition per line. * *P* < 0.05, ** *P* < 0.01, one-way ANOVA with Tukey and Dunnett multiple comparisons. **c** As (**b**) but after a regrowth period of 6–8 weeks after drug wash-out. **d** Brightfield images show the regenerative capacity of the organoids per condition, six to eight weeks after drug wash-out, before cell viability readout of (**c**). AC Adenocarcinoma, SRC Signet Ring Cell carcinoma, MC Mucinous Carcinoma. **e** as (**a**), six additional distinct PMDOs were treated with MMC (0.05-0.1 µM) or berzosertib (0.1–0.4 µM) alone and in combination for 72 h. Brightfield images show the regenerative capacity of the organoids per condition six weeks after drug wash-out. **f** As (**e**), but after a regrowth period of 6 weeks after drug wash-out. **g** Brightfield images show the regenerative capacity of the organoids per condition, six to eight weeks after drug wash-out, before cell viability readout of (**f**). Brightfield images are displayed in Figs. S3–4.

Next, we assessed the effect of berzosertib on short-term survival and long-term regrowth capacity (*i.e*., in vitro ‘recurrence’) of chemotherapy-treated PMDOs. To this end, 4 PMDOs derived from histopathologically distinct tumor types (adenocarcinoma, mucinous carcinoma, signet ring cell carcinoma) were treated with single drugs (MMC, oxaliplatin, irinotecan, berzosertib), and with the combinations of each chemotherapy and berzosertib. Three days after the start of treatment, half of the treated PMDOs were used to directly assess cell viability by measuring cellular ATP levels. The other half was used to determine regrowth potential after drug wash-out over a period of up to 8 weeks. Regrowth was assessed by microscopy over time and was quantified at the end of the experiments by measuring cellular ATP levels (Fig. 2a).

After a 3-day treatment with the single drugs (MMC, Oxaliplatin, irinotecan, berzosertib), cell viability was reduced to 63-110%, 26-47%, 15-38%, and 34-55% of control, respectively (Fig. 2b). The combination of Berzosertib with each chemotherapy further reduced cell viability to some extent in all 4 PMDOs, but this was not significantly different from the reduction caused by berzosertib alone (Fig. 2b).

Following drug wash-out, all PMDOs treated with any of the single drugs resumed growth within 8 weeks (Figs. 2c; S3), thus mimicking the clinical problem of recurrence. However, simultaneous exposure to MMC and berzosertib completely prevented regrowth in all 4 PMDOs (Fig. 2c, d). Treatment of PMDOs with oxaliplatin and berzosertib prevented regrowth in 2 out of 4 cases (Figs. 2c, d, S3). By contrast, treatment of PMDOs with irinotecan and berzosertib failed to prevent regrowth in any of the PMDOs, presumably because this combination also failed to augment DNA damage and replication stress (Fig. 1a).

The above results indicate that the combination of berzosertib and MMC is highly effective in preventing PMDO regrowth. To further test the potential efficacy of this specific drug combination, we performed direct readouts and long-term regrowth assays on 6 additional PMDOs derived from PM of diverse histopathological subtypes. Three-day treatment with MMC alone did not significantly reduce cell viability (Fig. 2e). Co-treatment with MMC and berzosertib for three days reduced cell viability to 27–81%. After drug wash-out, regrowth was observed in all 6 PMDOs following single drug treatment (MMC or berzosertib, Figs. 2f, g, S4). However, regrowth was completely prevented by the combination treatment in all but one PMDO, regardless of the histopathological subtype (Figs. 2f, g, S4).

To assess whether the effects of combination treatment were synergistic, we used all ATP measurements to calculate combination index (CI) values, which is a measure of synergy between two drugs [36, 37]. This revealed that MMC and berzosertib had additive or some synergistic effects on PMDO viability when measured immediately after treatment (i.e. 3 days, Table S3). Moreover, in all 10 PMDOs, MMC and berzosertib displayed synergy (CI < 0.5) or strong synergy (CI < 0.01) in preventing regrowth following drug wash-out (Table S3). No significant associations were found between mutation or prior treatment status and synergy score (CI value).

### ‘Adjuvant’ treatment with berzosertib prevents PMDO regrowth following in vitro HIPEC with MMC

**Fig. 3:**
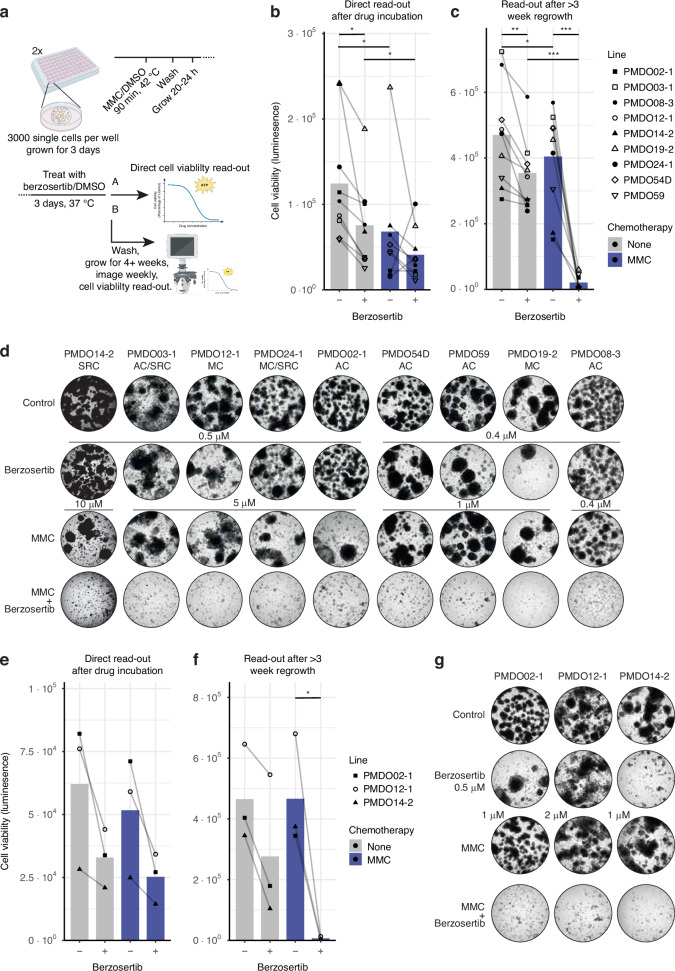
Berzosertib treatment prevents PMDO regrowth after 90-minute MMC treatment. **a** Experimental setup of the regrowth experiments. **b** Nine distinct PMDOs were treated for 90 minutes with MMC (0.4-10 µM) at 42 °C and 72 h berzosertib treatment (0.4–0.5 µM) after 20 h. Cell Titer Glo measurements were used to determine cell viability. Graphs show raw luminescence data. Each dot represents *n* ≥  3 per condition per line. * *P* < 0.05, ** *P* < 0.01, *** *P* < 0.001, one-way ANOVA with Dunnett and Tukey multiple comparisons. **c** As (**b**) but after a regrowth period of ≥24 days after berzosertib wash-out. **d** Brightfield images show the regenerative capacity of the organoids per condition ≥24 days after drug wash-out. AC Adenocarcinoma, SRC Signet Ring Cell carcinoma, MC Mucinous Carcinoma. **e** Three distinct PMDOs were treated for 90 min with MMC (1–2 µM) at 37 °C followed by 72 h berzosertib (0.5 µM) after 20 h. Cell Titer Glo measurements were used to determine cell viability. Graphs show the percentage of surviving cell fractions as % of untreated controls. * *P* < 0.05, one-way ANOVA with Dunnett and Tukey multiple comparisons **f** As (**e**) but after a regrowth period of 25 days after berzosertib wash-out. **g**. Brightfield images show the regenerative capacity of the organoids per condition, 25 days after drug wash-out. Brightfield images displayed in Fig. S6-7.

The above results demonstrate that berzosertib sensitizes a series of PMDOs to MMC regardless of the histological subtype. However, treatment duration in the above experiments was 1–3 days, while during HIPEC, MMC is administered for only 90 minutes. We reasoned that berzosertib might be effective as adjuvant treatment following HIPEC. To model this, we exposed 9 distinct PMDOs to ‘in vitro HIPEC’ consisting of a 90-minute exposure to MMC at 42 °C [27], and this was followed by a 3-day exposure to berzosertib. As above, we measured cell viability directly after berzosertib wash-out and subsequently followed regrowth for a period of 25 days by microscopy. At the end of the experiments, regrowth was quantified by ATP measurements (Fig. 3a).

First, we analyzed whether HIPEC-MMC caused ATR activation by performing a western blot of p-Chk1 over time following HIPEC. This revealed that ATR was activated 20-24 h after HIPEC (Fig. S5). Based on this result we started ‘adjuvant treatment’ with berzosertib 20 h following HIPEC for a period of 3 days.

Analysis of cell viability immediately after HIPEC +/- adjuvant treatment showed that HIPEC alone (MMC) reduced cell viability in all PMDOs and that this effect was highly variable (ranging from 10-90%; Fig. 3b). Berzosertib treatment alone (without HIPEC) reduced cell viability by 25-60%. Berzosertib treatment following HIPEC did not significantly alter cell viability when compared to HIPEC alone.

Microscopic analysis of regrowth over time showed that after drug wash-out, all 9 PMDOs rapidly resumed growth following single drug treatment with MMC (Fig. S6), again mimicking the clinical problem of recurrence. However, adjuvant berzosertib completely prevented regrowth following HIPEC in all 9 PMDOs (Fig. 3c, d). Analysis of ATP values revealed that HIPEC-MMC and adjuvant berzosertib displayed strong synergy (CI values < 0.01) in preventing regrowth (Table S4). No significant associations were found between mutation or prior treatment status and synergy score (CI value).

To assess the necessity of heat for the synergy of this combination, we repeated the HIPEC experiments exactly as described above with 3 PMDOs but with MMC treatment at 37 °C (NIPEC). Single treatments reduced cell viability measured immediately after drug wash-out (Fig. 3e). Combination treatment was more effective than MMC treatment alone but similar to berzosertib treatment alone (Fig. 3e). Regrowth after single drug treatment was observed in all cases but was completely prevented following combination treatment, again with strong synergy between drugs (Figs. 3f, g, S7, Table S4).

These results demonstrate that a short treatment with MMC at 37 °C, followed by ‘adjuvant treatment’ with berzosertib, is sufficient to prevent PMDO recurrence.

### DDR inhibitors prevent PMDO regrowth following MMC treatment

**Fig. 4:**
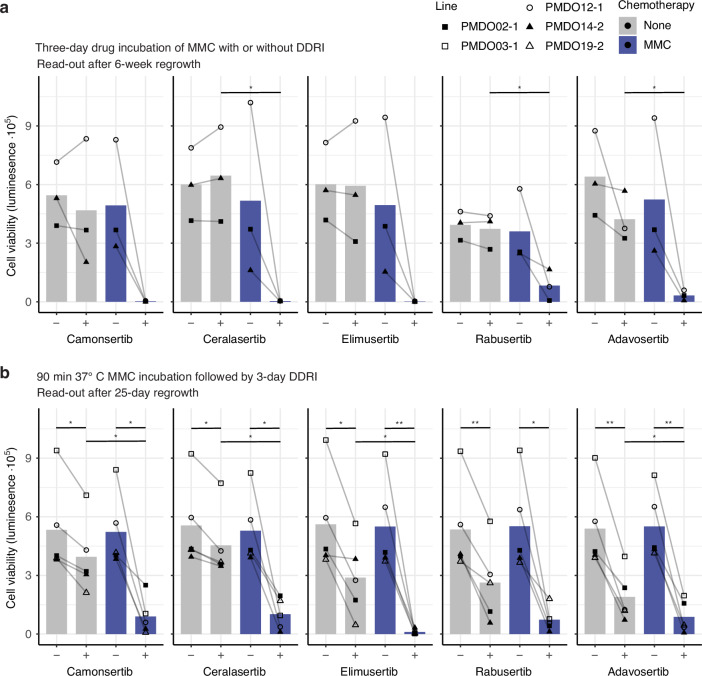
Additional clinically relevant DDR inhibitors tested also prevent PMDO regrowth after three-day and NIPEC MMC combination treatment. **a** Three distinct PMDOs were treated with MMC (0.1 µM), DDR inhibitor camonsertib, ceralasertib, elimusertib, rabusertib, or adavosertib (0.4–2 µM) alone or in combination for 72 h and allowed to regrow for 25 days, after which cell viability was measured using Cell Titer Glo. **b** Five distinct PMDOs were treated with 90 min 37 °C MMC (1–5 µM) and adjuvant 72 h DDR inhibitor camonsertib, ceralasertib, elimusertib, rabusertib, or adavosertib (0.1–5 µM) treatment or controls and allowed to regrow for 25 days, after which cell viability was measured using Cell Titer Glo. Graphs show raw luminescence data; each dot represents *n* ≥  3 per condition per line, and concentrations correspond to Tables S5-6. * *P* < 0.05, ** *P* < 0.01, one-way ANOVA with Dunnett and Tukey multiple comparisons. Brightfield images are displayed in Figs. S9-10.

To maximize the chance of clinical translation of this study, we tested whether other clinically relevant DDR inhibitors could sensitize PMDOs to MMC. To this end, we selected the ATR inhibitors camonsertib [39, 40], ceralasertib [41, 42], and elimusertib [43, 44], the Chk1 inhibitor rabusertib [45, 46], and the Wee1 inhibitor adavosertib [47]. Three PMDOs were exposed for three days to MMC (at 37 °C) alone, to each of the DDR inhibitors alone (3 days), or to the various combinations. Upon direct readout after 3 days, there were no significant effects of MMC + DDR treatment (Fig. S8). In some experiments, the combination significantly reduced cell viability compared to MMC alone but not compared to the DDR inhibitor alone (Fig. S8). However, the combination treatment with each of the DDR inhibitors completely or largely prevented regrowth over a period of 25 days (Figs. 4a, S9). Analysis of ATP measurements showed that, in each case, the MMC/DDR-inhibitor combination displayed strong synergy in preventing PMDO regrowth (CI < 0.2) (Table S5). Next, we also tested these inhibitors in the in vitro NIPEC setting by treating five PMDOs with MMC for 90 min at 37 °C and, after 20 h, adding the DDR inhibitors for 72 h. After wash-out and the 25-day regrowth period, control and monotherapy conditions showed viable PMDOs, whereas adjuvant DDR inhibition completely or largely prevented regrowth (Figs. 4b, S10). Analysis of ATP measurements showed that in each case, the MMC/DDR-inhibitor combination displayed synergy or strong synergy in preventing PMDO regrowth (CI < 0.8) (Table S6).

## Discussion

The combination of DDR inhibitors with chemotherapy is actively being explored as a potentially effective anti-cancer strategy [14, 17, 18]. In this report, we show that inhibition of the DDR using various clinically relevant drugs, prevents the regrowth of a large panel of organoids derived from peritoneal metastases following a 90-minute treatment with MMC, as is routinely performed during the HIPEC procedure. Therefore, adjuvant treatment with DDR inhibitors may be an effective strategy to prevent local intraperitoneal recurrence following HIPEC with MMC.

Multiple inhibitors of the DDR are currently being tested clinically, which gives room for a variety of clinical study designs [20]. The focus of this study was on berzosertib, which is a well-tolerated first-in-class ATR inhibitor that is currently in Phase II trials [48–50]. However, Merck KGaA has begun shifting its focus from berzosertib to tuvusertib (M1774), a newer, orally administered ATR inhibitor in clinical development [51]. To assess the general applicability and translatability of the approach, we used additional ATR inhibitors camonsertib, ceralasertib, and elimiusertib. Camonsertib is being tested in Phase I, where preliminary results showed tolerability and anti-tumor activity [52]. Ceralasertib is in Phase II trials, is well-tolerated, has shown effectiveness, and might be more selective than berzosertib [43, 50]. Elimusertib is in Phase I trials and shows tolerability and response in multiple cancers with ATM aberrations [53]. We also used rabusertib, which is a Chk1 inhibitor currently in Phase II trials. Unfortunately, rabusertib has not shown an added effect on chemotherapeutics in patients with the tumor types tested [54, 55]. This has led to a shift of clinical focus to Chk1 inhibitor prexasertib [56]. Lastly, we tested adavosertib, which targets the DDR indirectly as a Wee1 inhibitor that leads to cell cycle progression through activated CDK1 [57, 58]. Adavosertib has been tested in phase I and II trials but showed poor tolerability, and the development of this drug has been discontinued [59]. From the above studies, it is hard to extrapolate which DDR inhibitor is most likely to be effective in the treatment of PM from CRC. Besides, new drugs are continuously being developed and clinically tested, making it a rapidly evolving field [51, 60, 61]. Additionally, factors such as safety profile, patient convenience (e.g., route and frequency of administration), and cost-effectiveness will also play an important role in determining which agents may ultimately be viable for clinical use.

Based on the CI scores of the PMDO screens, Elimusertib and Berzosertib seem most promising for PM-CRC. Nevertheless, the in vitro concentrations of, for example, Ceralasertib could be optimized since we did not reach concentrations that killed all or most PMDOs in monotherapy. However, clinical concentrations should be assessed in a trial, as both tolerability and efficacy are different in patients compared to preclinical in vivo and in vitro models. Moreover, clinical studies are needed to assess whether adjuvant treatment with DDR inhibitors is best given intraperitoneally, for instance, using a port-a-cath, or whether systemic treatment would suffice.

As HIPEC is preceded by surgical removal of all visible tumor deposits, the clinical endpoints of an adjuvant study should be either time-to-(local) recurrence or survival. To de-risk such a study, it may be attractive to first perform a smaller proof-of-concept study in patients with inoperable disease, for instance, in patients treated with pressurized intraperitoneal aerosol chemotherapy (PIPAC). The results of a first PIPAC study employing MMC are eagerly awaited [62], as they may provide a basis for safely combining PIPAC-MMC with DDR inhibitors. A major advantage of such a study would be the possibility to monitor treatment effects ‘online’ given the cyclic nature of the procedure, which allows repeated access to the peritoneal cavity over time. In this way, biological in-patient proof-of-concept could be obtained for the effectiveness of the combination therapy.

Notably, intraperitoneal administration may allow for higher dose tolerability compared to systemic delivery, potentially contributing to treatment success. We previously reported that MMC concentrations during HIPEC procedures range from ~10–20μM [27], exceeding the minimal effective doses in our in vitro models and supporting the clinical relevance of our chosen concentration. However, for berzosertib or other DDR inhibitors, intraperitoneal administration has not been clinically explored, and intratumoral concentrations following IV delivery remain unknown. However, clinical trials have shown plasma levels up to 2.3 µM at tolerable doses [49]. While direct comparisons between in vitro and clinical settings are limited by differences in drug distribution, metabolism, and the tumor microenvironment, available clinical and preclinical data would suggest that our in vitro concentration of 0.4 µM is within a plausible range and provides a rational starting point for further investigation.

Furthermore, our results show that heat was not necessary for the synergistic killing of PMDOs by MMC and berzosertib. Heat can augment the cytotoxic effects of multiple chemotherapeutic drugs on cancer cell viability, but the added effect of heat in HIPEC is debated and understudied [63–65]. Of note, one study showed that, in vitro, MMC is active independent of heat and that the synergism of heat and MMC was cell type-dependent [66]. Specifically, they showed that MMC treatment on CRC cell lines led to increased apoptosis, G2-arrest, and decreased cell growth at both 37 and 42 °C, and that there was no synergy with heat.

Aside from MMC, oxaliplatin and irinotecan are also used for intraperitoneal treatment of PM-CRC [8–11]. MMC and oxaliplatin are alkylating agents that cause direct DNA damage [67]. Irinotecan is an inhibitor of topoisomerase I, causing indirect DNA damage in the form of double-strand DNA breaks [68–70]. The synergy between oxaliplatin and berzosertib was observed in only a subset of PMDOs. In addition, the combination of irinotecan with berzosertib failed to prevent regrowth of all tested PMDOs. By contrast, the synergy between MMC and DDR inhibitors was observed for all PMDOs. The observed drug-dependent synergy with DDR inhibitors may be due to differential DDR activation by the different chemotherapies. Drug resistance is a complex and often multifactorial process, influenced by a diversity of molecular mechanisms [71–73]. For instance, it has been shown that oxaliplatin induces ribosome biogenesis stress rather than stress that triggers the DDR [74, 75]. For instance, the glutathione pathway detoxifies oxaliplatin [28, 76] and prevents the induction of DNA damage. If such resistance mechanisms are dominant, the combination with DDR inhibitors is unlikely to be effective. Likewise, resistance to irinotecan can occur through upregulation of ABC transporters for drug efflux, a decreased level of Topoisomerase I, or single nucleotide polymorphisms (SNPs) in the TOP1 gene that prevent SN-38 from creating a stable bond [77, 78]. These are all mechanisms that prevent DNA damage induction in the first place. Therefore, after exposure to irinotecan or oxaliplatin, DDR activation is less likely to be important for tumor cell survival. Thus, to develop synergistic combination treatment strategies with oxaliplatin and irinotecan, the most dominant resistance mechanisms that protect PM from these drugs need to be identified.

In conclusion, our study forms a solid basis for exploring the clinical value of adjuvant treatment with DDR inhibitors following MMC-based HIPEC, in order to limit recurrence. The uniform response of a heterogeneous series of 10 PMDOs harboring distinct genetic drivers, from different histopathological subtypes, and with different treatment histories, suggests that patient selection in such clinical studies may not be necessary.

## Supplementary information

Supplemental tables

Supplementary figures

## Data Availability

The datasets used and/or analyzed during this study are available from the corresponding author on reasonable request.
