# Supplementary material for: Targeting the DNA damage response prevents regrowth of colorectal peritoneal metastasis-derived organoids following treatment with mitomycin C
Source: Br J Cancer. 2026 Jan 5;134(5):831–42. doi: 10.1038/s41416-025-03310-z (PMC12905249; doi:10.1038/s41416-025-03310-z)
Supplement: Supplementary file 1 — Supplemental tables [file 41416_2025_3310_MOESM1_ESM.pdf]

Supplementary table 1. Organoid and matching patient details.

Organoid ID	location PM	Gender	Age at inclusion	TNM stage at diagnosis	Location primay CRC	Histology	MSI-Status	Chemo naïve sample	Therapies seen	Included under	Previous publications
PMDO02-1	ascites	M	57	pT2N1M0	Hepatic flexure	AC	MSS	yes	-	PIPAC-I	Ubink et al, 2019 BJS; Laoukili et al, 2022 BJC
PMDO03-1	ascites	M	43	cT4N1M0	caecum	AC/SRC	MSS	yes	-	PIPAC-I	Laoukili et al, 2022 BJC
PMDO08-3	ascites	F	78	unknown	right colon	AC	MSS	no	Oxaliplatin	PIPAC-I	Laoukili et al, 2022 BJC
PMDO12-1	ascites	M	75	pT3N1M0	sigmoid	MC	unknown	no	5-FU, Bevacizumab, Irinotecan, Oxaliplatin	PIPAC-I	
PMDO14-2	ascites	F	66	cTxNxM1	sigmoid	SRC	unknown	no	Capecitabine, Bevacizumab, Oxaliplatin	PIPAC-I	Laoukili et al, 2022 BJC
PMDO19-2	ascites	F	60	cT3N0M1	sigmoid	MC	unknown	no	Capecitabine, 5-FU, Bevacizumab, Irinotecan, Oxaliplatin	PIPAC-I	Laoukili et al, 2022 BJC
PMDO20-1	ascites	F	68	pT2N0M0	sigmoid	MC	MSS	yes	-	PIPAC-I	
PMDO24-1	ascites	F	70	cT3N1M1	transversum	MC/SRC	unknown	yes	-	PIPAC-II	
PMDO054D	peritoneum at liver	F	72	T4N0M1	colon ascendes	AC	MSS	yes	-	ORCAPM	
PMDO059	ascites	M	69	T4N1M0	Rectum	AC	unknown	no	5-FU, Bevacizumab, Irinotecan, Oxaliplatin	INTERACT-II	

Supplementary table 2. Mutation data of organoid based on WGS/TSO500 sequencing for selected genes. Only class 4 and 5 (bold) mutations annotated. Matching patient clinically known mutations noted.

	**PMDO02-1**	**PMDO03-1**	**PMDO08-3**	**PMDO12-1**	**PMDO14-2**	**PMDO19-2**	**PMDO20-1**	**PMDO24-1**	**PMDO054D**	**PMDO059**
MSS/MSI			MSS		MSS	MSI		MSS	MSS	MSS
APC	**W1049***	K1555fs		**Q1367***, K758fs	**E1554***	**E1464Vfs*8**	K1555fs		N1455Ifs*18	**R283***
TP53	**Splice**	**Splice**			K132N	**R273C**, S215G			Splice	**R175H**
KRAS				**G13D**					**G12D**	A146V
BRAF			**V600E**			**V600E**	**V600E**	**V600E**		
PIK3CA		M1043V			M1043V					
FBXW7				R278*, R224*					T271*	R425C
ATM										
NRAS										
SMAD4			**R361S**	R361C			R361H	**R361S**		
TCF7L2										
MSH3							V891fs, M892fs			
**Clinically reported patient mutations:**			**BRAF** V600E, **SMAD4** R361S	**APC** Q1367*, **KRAS** G13D, **FBXW7** R278*, **SMAD4** R361C				**BRAF** mt	**KRAS** mt	

Supplementary table 3. Combination index for direct and regrowth (≥6 weeks) after three-day drug screen with MMC and berzosertib

Organoid line	MMC (μM)	Berzosertib (μM)	Combination index direct readout	Direct synergy class	Combination index regrowth readout	Regrowth synergy class
PMDO14-2	0.10	0.4	0.81	Add	0.04	SS
PMDO02-1	0.10	0.4	1.12	Add	0.01	SS
PMDO24-1	0.10	0.4	0.68	S	0.01	SS
PMDO08-3	0.10	0.4	0.84	Add	0.01	SS
PMDO059	0.10	0.4	0.23	SS	0.42	SS
PMDO19-2	0.10	0.4	0.27	SS	0.00	SS
PMDO03-1	0.10	0.4	0.62	S	0.01	SS
PMDO12-1	0.10	0.4	0.71	S	0.01	SS
PMDO054D	0.05	0.4	0.83	Add	0.01	SS
PMDO20-1	0.05	0.4	0.80	Add	0.01	SS

Sub-additive (SA) CI > 1.2; additive (Add) CI 0.8-1.2; synergistic (S) CI < 0.8; strong synergistic (SS) CI < 0.5.

Supplementary table 4 - part 1. Combination index for direct and regrowth (≥24 days) after *in vitro* HIPEC (90 min MMC at 42°C) screen with three day adjuvant berzosertib

Organoid line	MMC (µM)	Berzosertib (µM)	Combination index direct readout	Direct synergy class	Combination index regrowth readout	Regrowth synergy class
PMDO14-2	10.0	0.5	1.56	SA	0.04	SS
PMDO03-1	5.0	0.5	0.84	Add	0.02	SS
PMDO12-1	5.0	0.5	5.88	SA	0.01	SS
PMDO24-1	5.0	0.5	8.20	SA	0.03	SS
PMDO02-1	5.0	0.5	1.44	SA	0.04	SS
PMDO054D	1.0	0.4	1.10	Add	0.02	SS
PMDO059	1.0	0.4	0.60	S	0.19	SS
PMDO19-2	1.0	0.4	0.73	S	0.03	SS
PMDO08-3	0.4	0.4	0.61	S	0.01	SS

Supplementary table 4 - part 2. Combination index for direct and regrowth (25 days) after in vitro NIPEC (90 min MMC at 37°C) screen with three day adjuvant berzosertib

Organoid line	MMC (µM)	Berzosertib (µM)	Combination index direct readout	Direct synergy class	Combination index regrowth readout	Class
PMDO02-1	1.0	0.5	0.93	Add	0.03	SS
PMDO12-1	2.0	0.5	1.00	Add	0.02	SS
PMDO14-2	1.0	0.5	0.78	S	0.03	SS

Sub-additive (SA) CI > 1.2; additive (Add) CI 0.8-1.2; synergistic (S) CI < 0.8; strong synergistic (SS) CI < 0.5.

Supplementary table 5. Combination index for regrowth (25 days) after three-day drug screen with MMC and DDRi

Camonsertib

Organoid line	MMC (µM)	Camonsertib (µM)	Combination index direct readout	Direct synergy class	Combination index regrowth readout	Regrowth synergy class
PMDO02-1	0.1	2.0	1.24	SA	0.01	SS
PMDO12-1	0.1	0.1	0.78	S	0.01	SS
PMDO14-2	0.1	0.4	0.77	S	0.01	SS

Ceralasertib

Organoid line	MMC (µM)	Ceralasertib (µM)	Combination index direct readout	Direct synergy class	Combination index regrowth readout	Regrowth synergy class
PMDO02-1	0.1	2.0	0.92	Add	0.01	SS
PMDO12-1	0.1	2.0	0.63	S	0.00	SS
PMDO14-2	0.1	0.4	0.90	Add	0.01	SS

Elimusertib

Organoid line	MMC (µM)	Elimusertib (µM)	Combination index direct readout	Direct synergy class	Combination index regrowth readout	Regrowth synergy class
PMDO02-1	0.1	0.4	1.39	SA	0.00	SS
PMDO12-1	0.1	0.1	0.60	S	0.00	SS
PMDO14-2	0.1	0.1	0.47	SS	0.01	SS

Rabusertib

Organoid line	MMC (µM)	Rabusertib (µM)	Combination index direct readout	Direct synergy class	Combination index regrowth readout	Regrowth synergy class
PMDO02-1	0.1	2.0	1.53	SA	0.03	SS
PMDO12-1	0.1	2.0	0.72	S	0.14	SS
PMDO14-2	0.1	0.4	0.55	S	0.01	SS

Adavosertib

Organoid line	MMC (µM)	Adavosertib (µM)	Combination index direct readout	Direct synergy class	Combination index regrowth readout	Regrowth synergy class
PMDO02-1	0.1	2.0	1.30	SA	0.12	SS
PMDO12-1	0.1	2.0	0.70	S	0.15	SS
PMDO14-2	0.1	0.1	0.84	Add	0.03	SS

Sub-additive (SA) CI > 1.2; additive (Add) CI 0.8-1.2; synergistic (S) CI < 0.8; strong synergistic (SS) CI < 0.5.

Supplementary table 6. Combination index for regrowth (25 days) after NIPEC screen (90min, 37°C) with MMC followed by three-day DDRi treatment

Camonsertib

Organoid line	MMC (µM)	Camonsertib (µM)	Combination index	Class
PMDO02-1	1.0	2.0	0.78	S
PMDO03-1	5.0	2.0	0.16	SS
PMDO12-1	2.0	0.1	0.14	SS
PMDO14-2	1.0	0.1	0.09	SS
PMDO19-2	1.0	0.4	0.03	SS

Ceralasertib

Organoid line	MMC (µM)	Ceralasertib (µM)	Combination index	Class
PMDO02-1	1.0	5.0	0.55	S
PMDO03-1	5.0	2.0	0.14	SS
PMDO12-1	2.0	5.0	0.09	SS
PMDO14-2	1.0	5.0	0.03	SS
PMDO19-2	1.0	2.0	0.48	SS

Elimusertib

Organoid line	MMC (µM)	Elimusertib (µM)	Combination index	Class
PMDO02-1	1.0	2.0	0.01	SS
PMDO03-1	5.0	2.0	0.00	SS
PMDO12-1	2.0	0.4	0.04	SS
PMDO14-2	1.0	0.1	0.09	SS
PMDO19-2	1.0	0.4	0.08	SS

Rabusertib

Organoid line	MMC (µM)	Rabusertib (µM)	Combination index	Class
PMDO02-1	1.0	5.0	0.34	SS
PMDO03-1	5.0	2.0	0.13	SS
PMDO12-1	2.0	5.0	0.16	SS
PMDO14-2	1.0	5.0	0.23	SS
PMDO19-2	1.0	2.0	0.70	S

Adavosertib

Organoid line	MMC (µM)	Adavosertib (µM)	Combination index	Class
PMDO02-1	1.0	2.0	0.63	S
PMDO03-1	5.0	2.0	0.55	S
PMDO12-1	2.0	5.0	0.24	SS
PMDO14-2	1.0	5.0	0.09	SS
PMDO19-2	1.0	2.0	0.35	SS

Sub-additive (SA) CI > 1.2; additive (Add) CI 0.8-1.2; synergistic (S) CI < 0.8; strong synergistic (SS) CI < 0.5.
