# Supplementary material for: Targeting the DNA damage response prevents regrowth of colorectal peritoneal metastasis-derived organoids following treatment with mitomycin C
Source: Br J Cancer. 2026 Jan 5;134(5):831–42. doi: 10.1038/s41416-025-03310-z (PMC12905249; doi:10.1038/s41416-025-03310-z)
Supplement: Supplementary file 2 — Supplementary figures [file 41416_2025_3310_MOESM2_ESM.pdf]

Supplementary figure 1

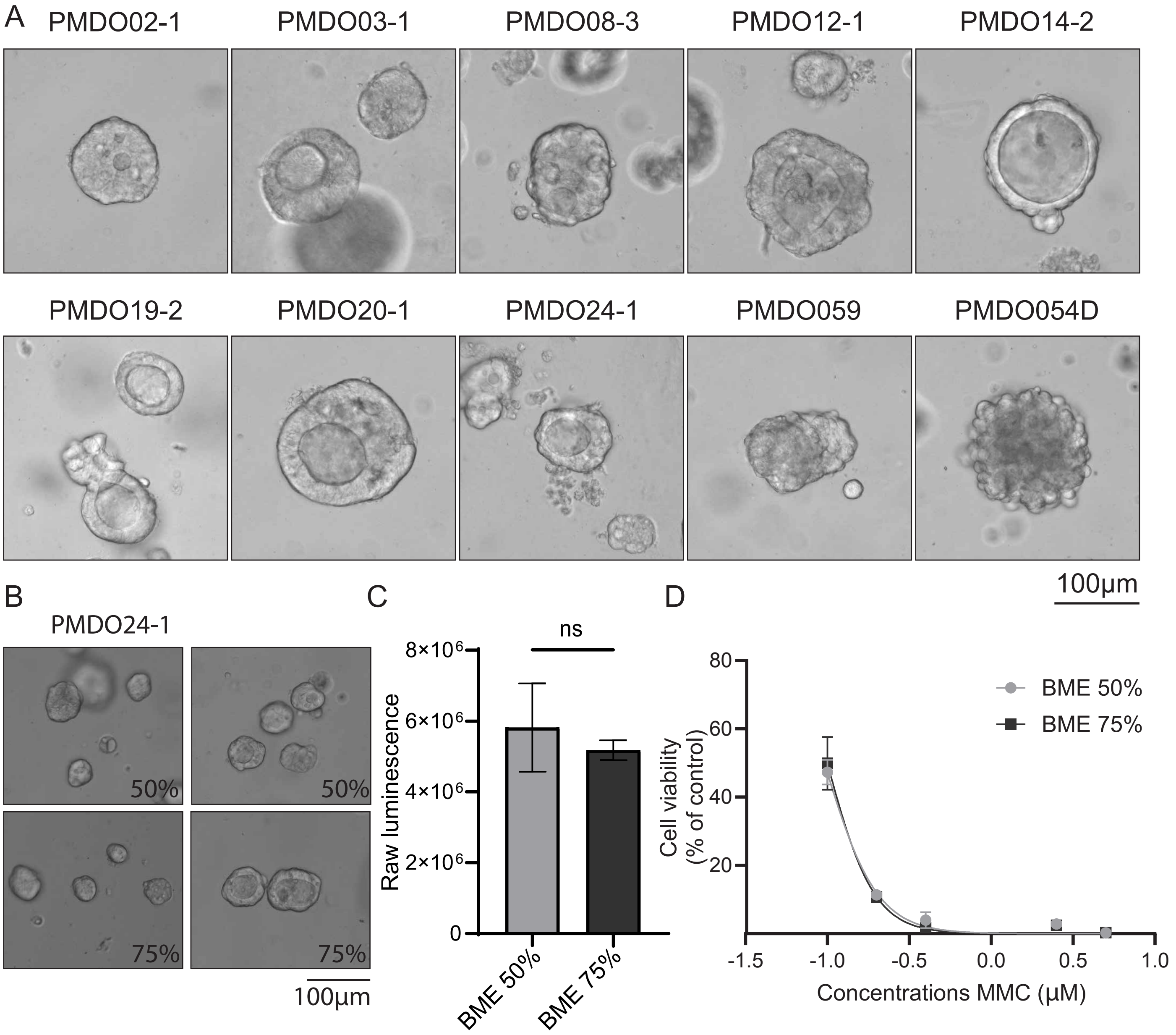

SF1. PMDOs from different PM-CRC patients exhibit similar yet distinct morphologies, with BME concentration having no effect on cell growth or drug response.
A. Brightfield images of ten-day-old organoids taken at the digital inverted microscope (EVOS) at a magnification of 20x. **B**. 20x images of PMDO24-1 grown in 50% or 75% BME. **C**. Cell viability of PMDO24-1 grown for eigth days in 50% or 75% BME. **D**. Dose-response curves of PMDO24-1 after five-day treatment of MMC in 50% or 75% BME.

Supplementary figure 2

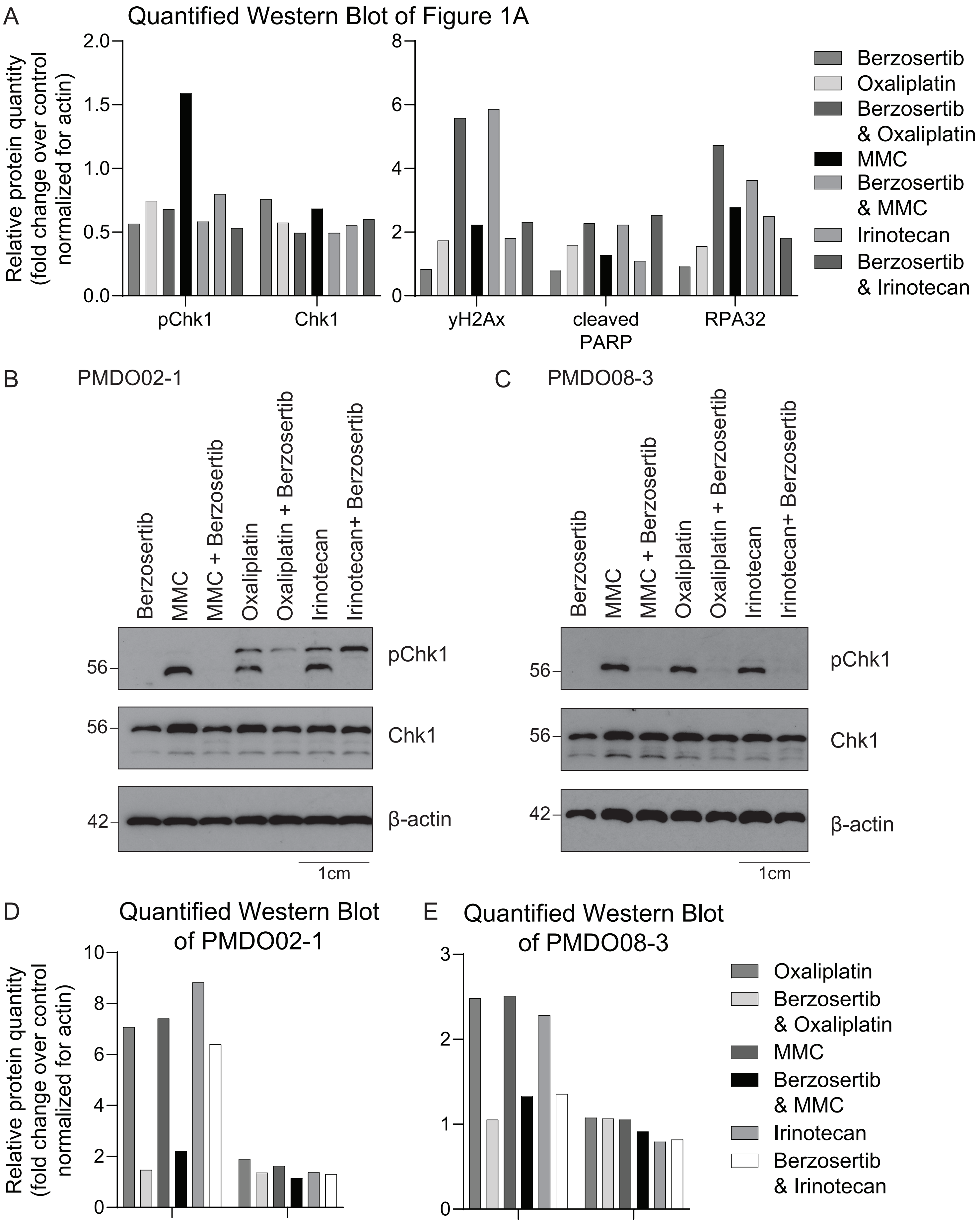

Supplementary figure 2

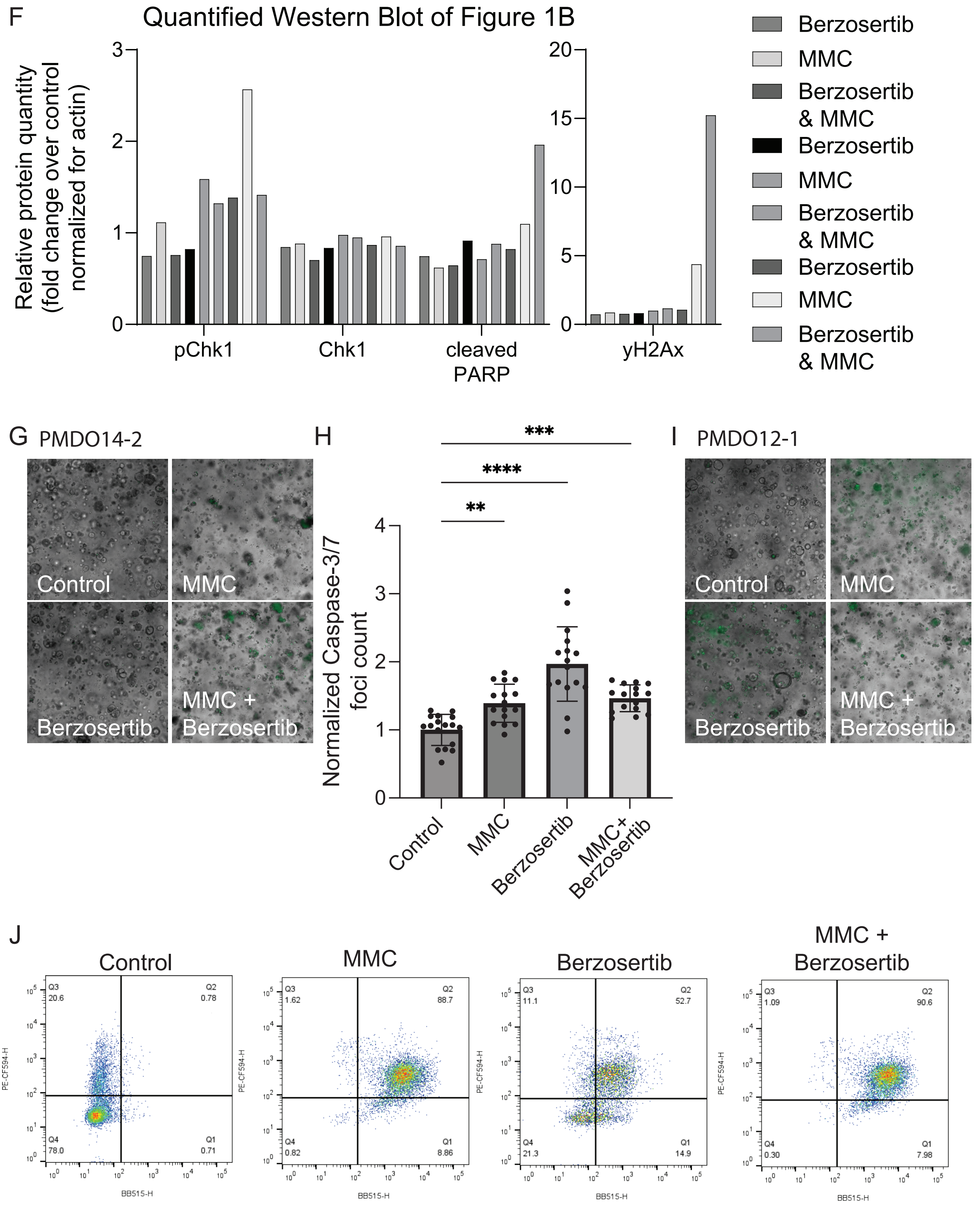

Supplementary figure 2

SF2. MMC, oxaliplatin, and irinotecan treatment induce pChk1 protein expression, which can be inhibited by berzoserib.

A. Quantification of Western blot of (**Fig. 1A**). **B.** Western blot analysis of pChk1 (Ser345), Chk1, and β-actin protein expression levels in PMDO02-1 organoids following exposure to MMC (10μM), oxaliplatin (220μM), or irinotecan (85μM), and berzosertib (5μM) or the combination for 4 hours. **C.** Treatment of PMDO08-3 as in (**C**). **D.** Quantification of Western blot of (**C**). **E.** Quantification of Western blot of (**D**). **F.** Quantification of Western blot of (**Fig. 1B**). **G.** Brigthfield images of PMDO14-2 after 3 day treatment with Caspase-3/7 (AF488) signal overlay as quantified in (**Fig. 1E**). **H.** Quantification of Caspase-3/7 signal in PMDO12-1 after 3-day treatment with MMC (10μM), berzosertib (5μM), or the combination. ** P<0.01, *** P<0.001, **** P<0.0001, one-way ANOVA with Tukey multiple comparisons. **I.** Brigthfield images of PMDO12-1 after 3 day treatment with Caspase-3/7 (AF488) signal overlay as quantified in (**I**). **J.** Density plots of the flow cytometry results quantified in (**Fig. 1F**).

Supplementary figure 3

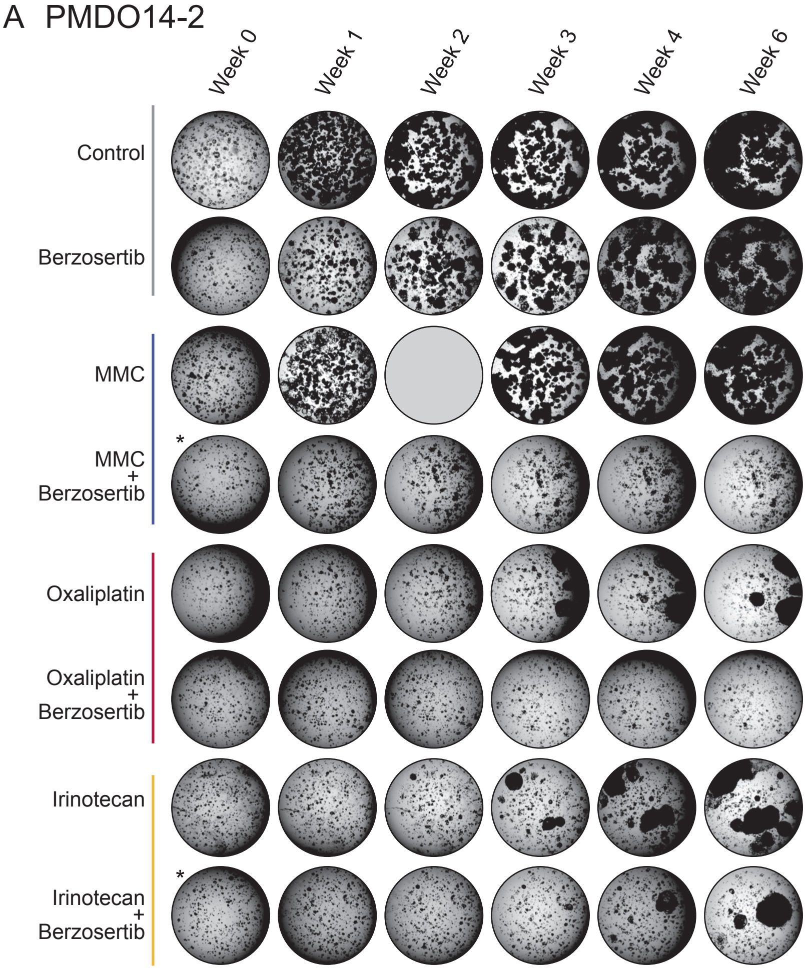

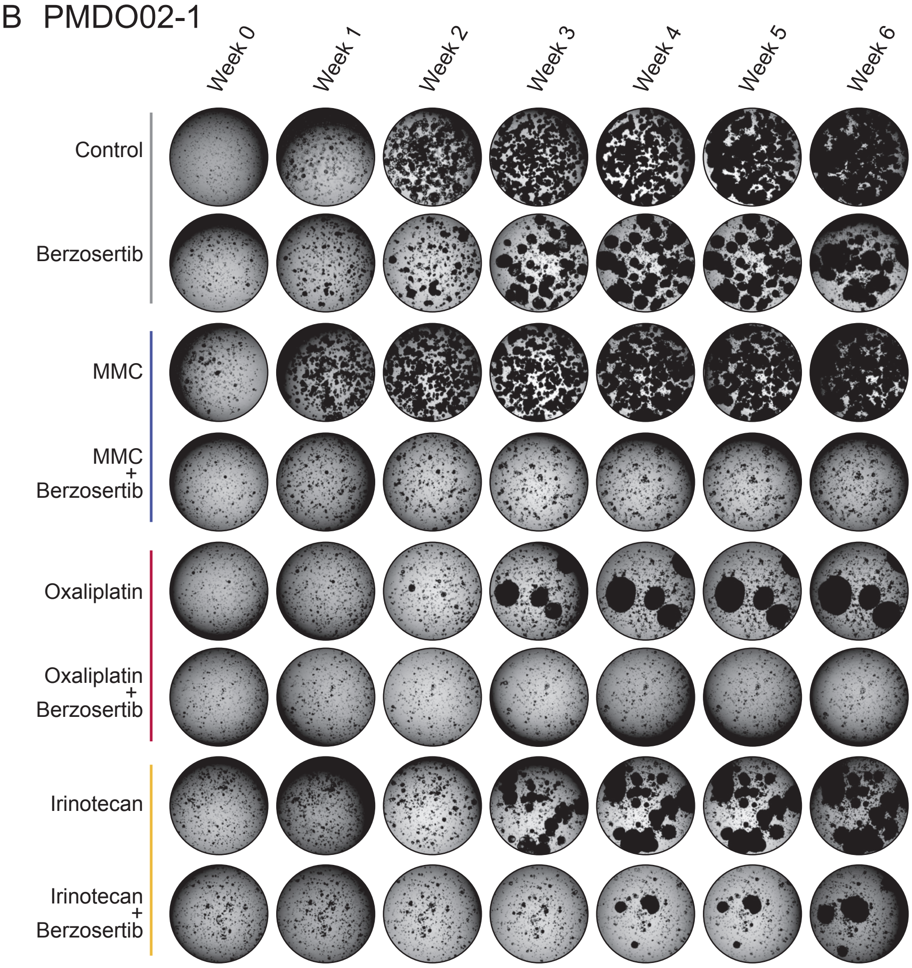

Supplementary figure 3

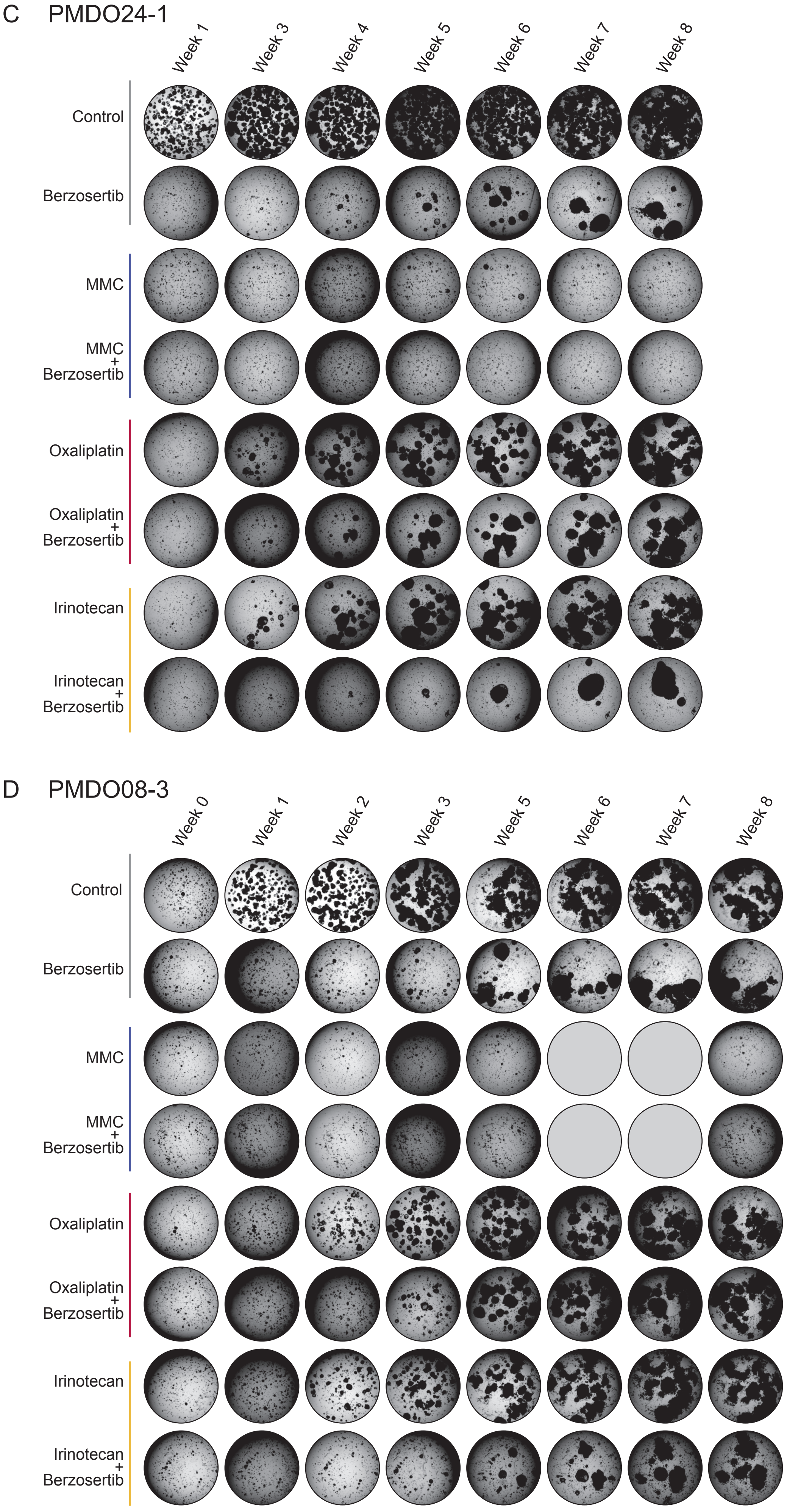

Supplementary figure 3

SF3. Regrowth assays show synergistic effects between MMC and berzosertib. Three-day MMC-berzosertib combination treatment prevents PM-CRC organoid regrowth

A. Photographic images of one example well per treatment condition of PMDO14-2 after 72-hour treatment with 0.1µM MMC, 60µM oxaliplatin, or 85µM irinotecan, and 0.4µM berzosertib alone and in combination over time of regrowth after drug washout. **B.** as in (**A**) for PMDO02-1. **C.** as in (**A**) for PMDO24-1 with 20µM oxaliplatin or irinotecan. **D.** as in (**C**) for PMDO08-3. *Indicates photo was taken from a replicate but not the same well as series.

Supplementary figure 4

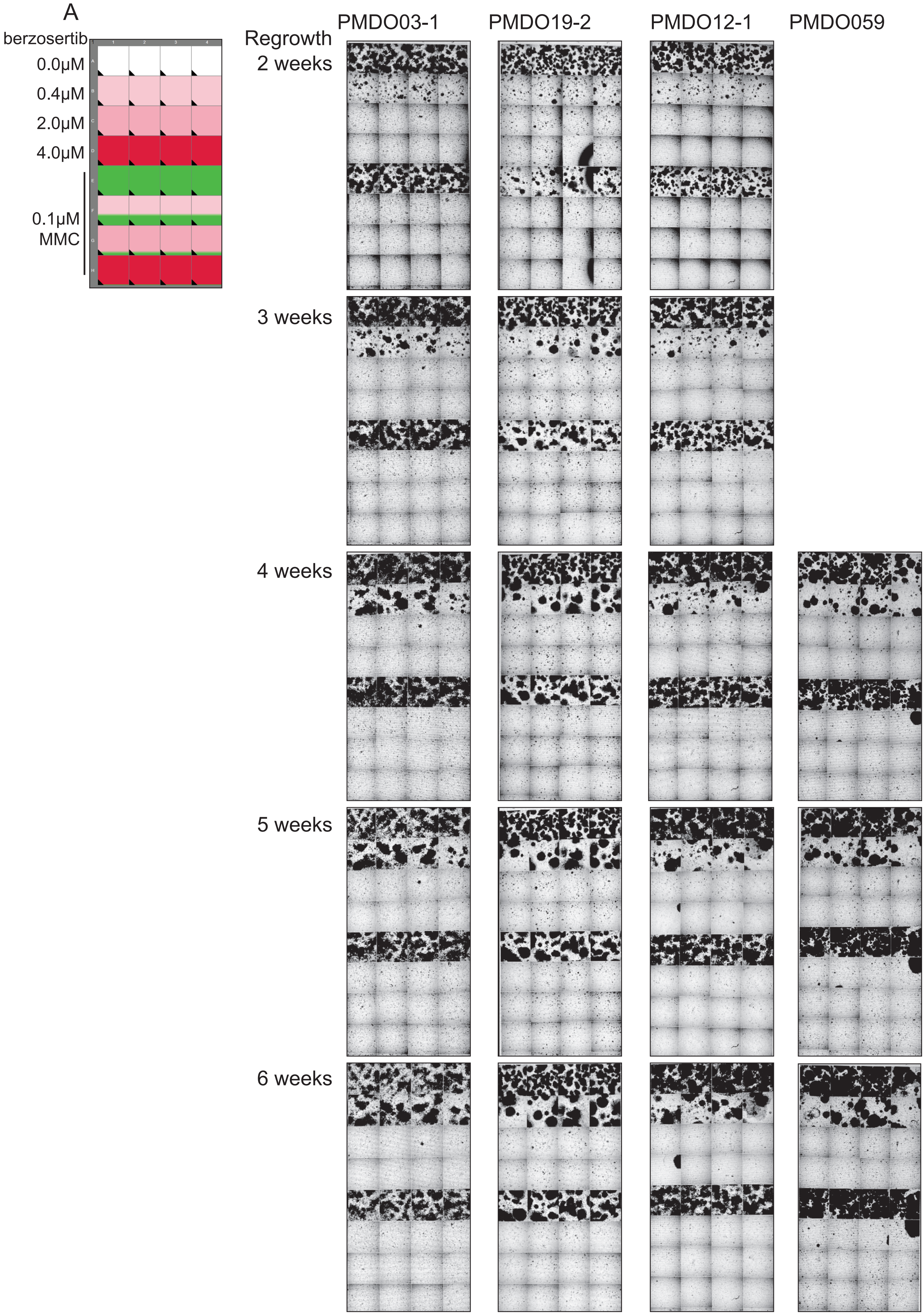

Supplementary figure 4

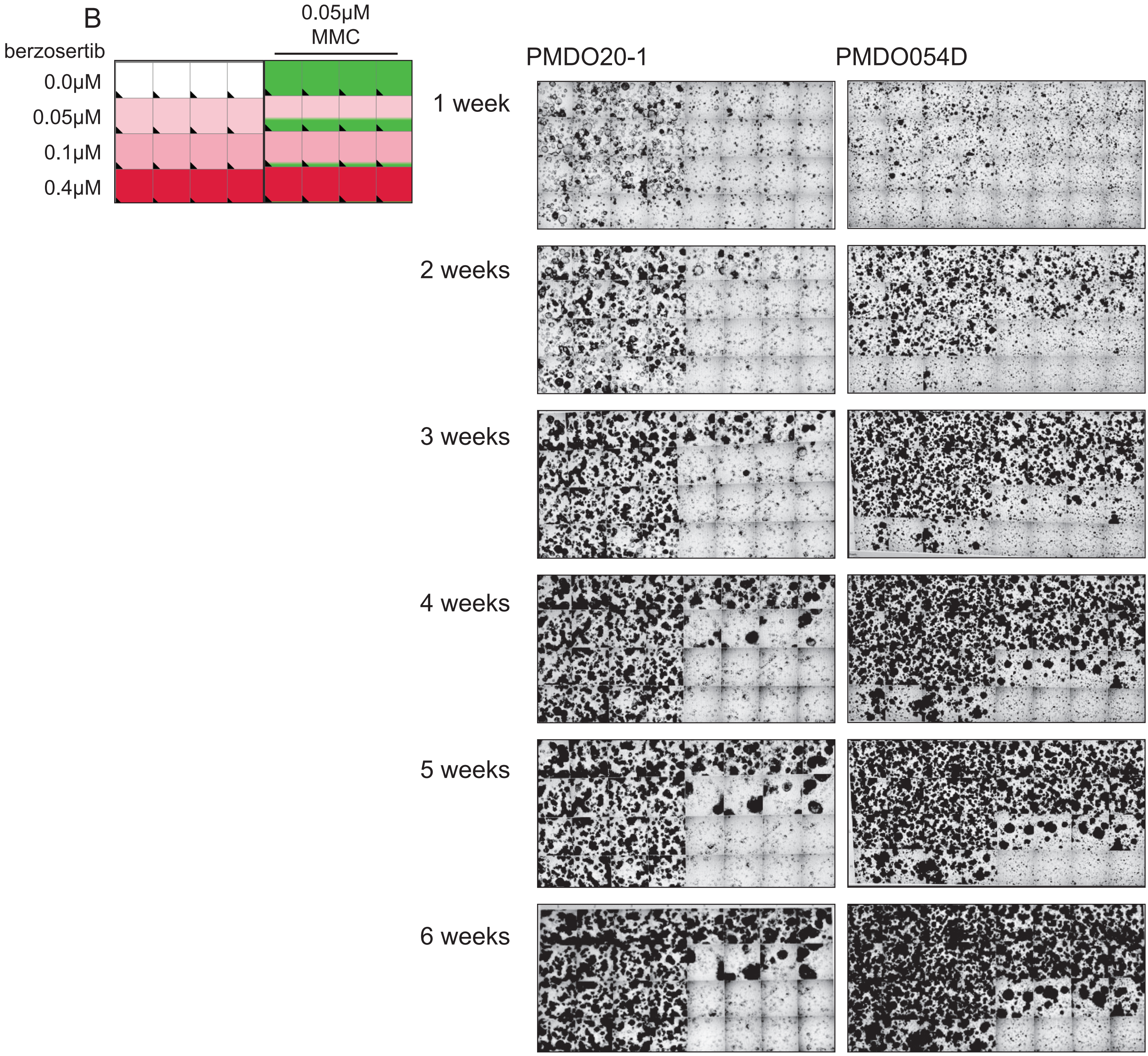

SF4. Outgrowth assays show synergistic effects between MMC and berzosertib.
A. Drug screen plate layout of 72 hour treatment and photographic images of the drug screen plates over time of regrowth after drug washout. **B.** as (**A**).

Supplementary figure 5

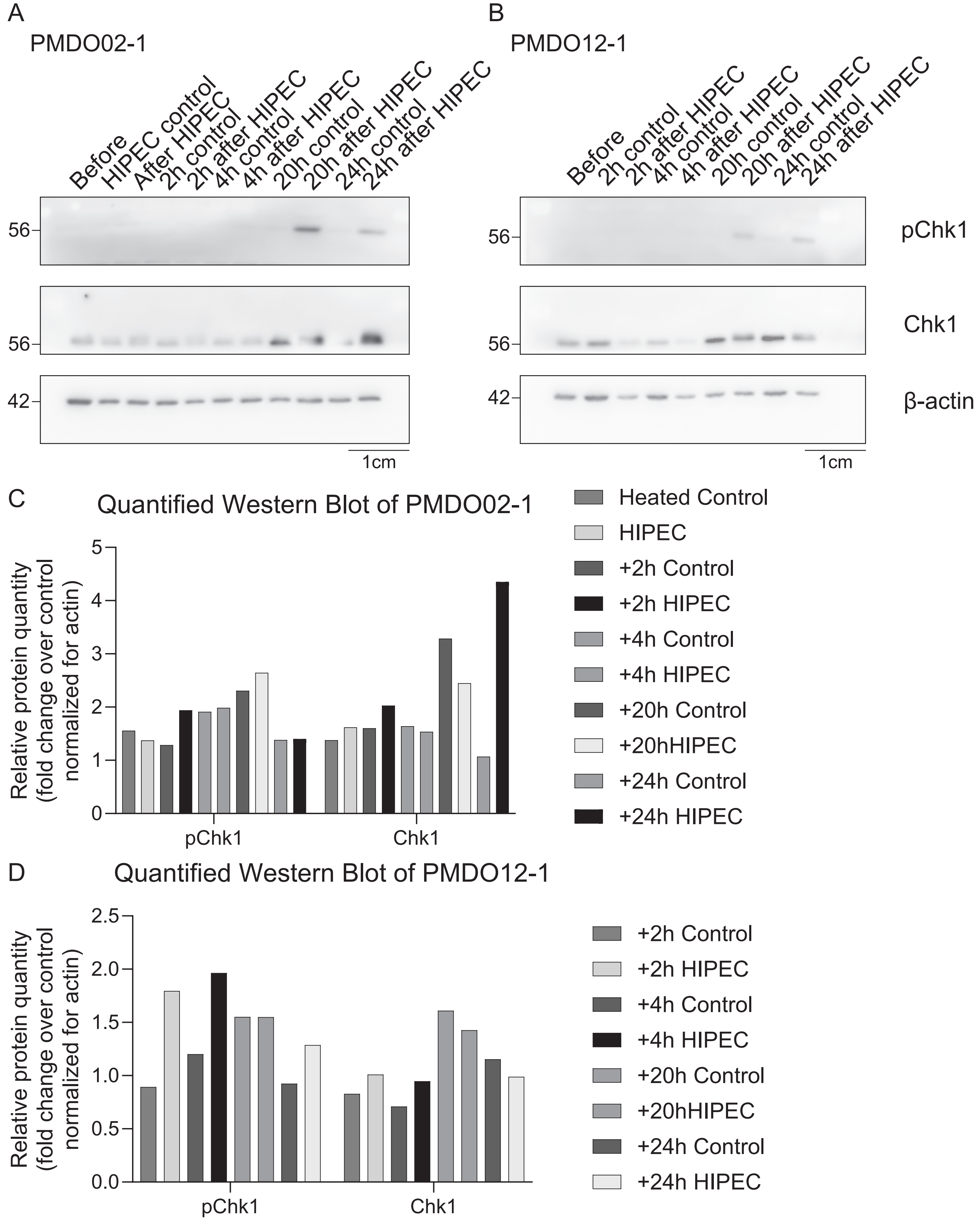

SF5. pChk1 protein expression is induced approximately 20 hours after 42°C 90-minute MMC treatment.

A. Western blot analysis of pChk1 (Ser345), Chk1, and β-actin protein expression levels in PMDO02-1 organoids following exposure to 10μM MMC for 90 minutes at 42°C harvested at the indicated time points. **B.** as (**A**) but for PMDO12-1. **C.** Quantification of Western blot of (**A**). **D.** Quantification of Western blot of (**B**).

Supplementary figure 6

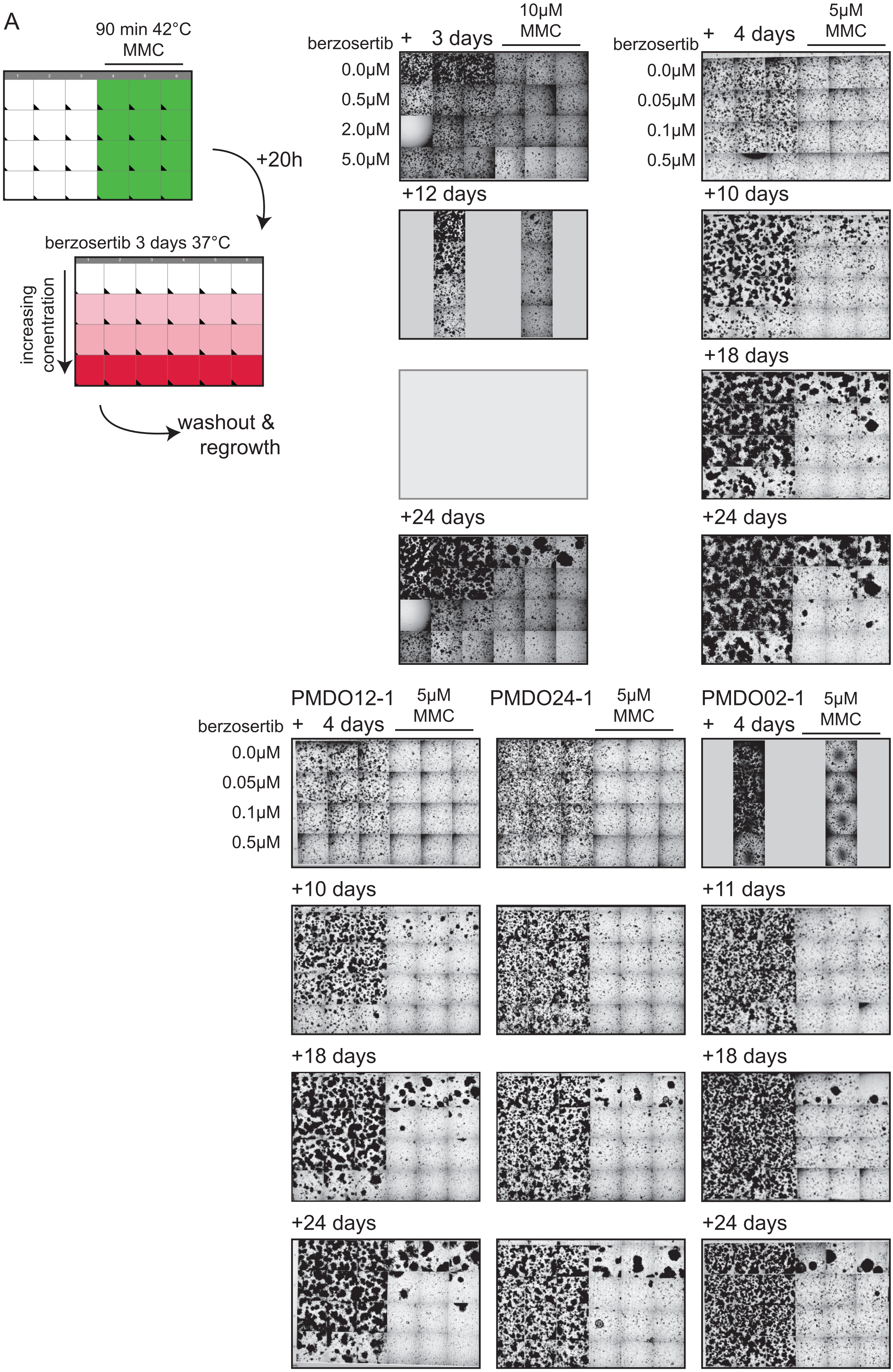

Supplementary figure 6

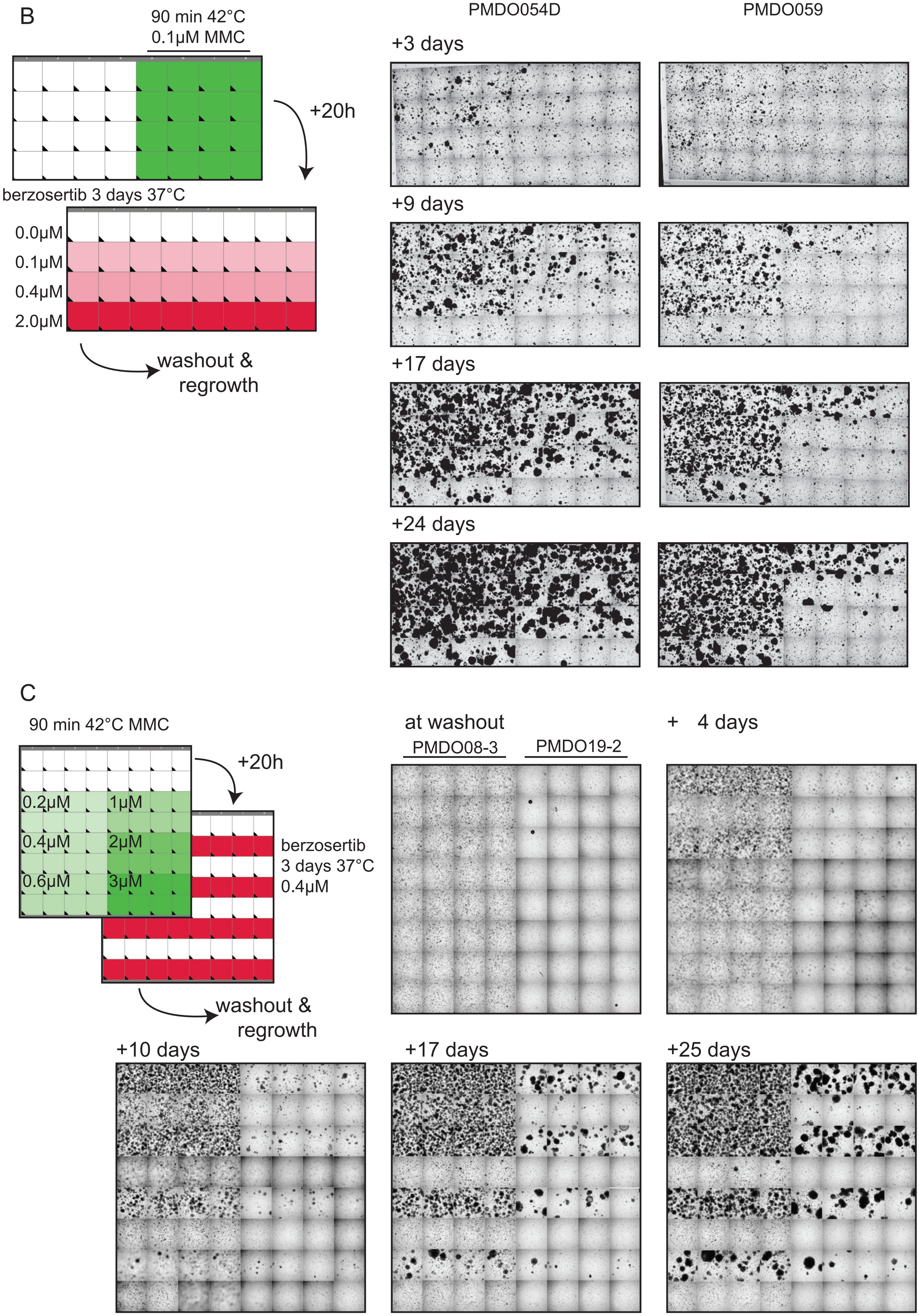

Supplementary figure 6

SF6. Outgrowth assays show synergistic effects of 90-minute 42°C MMC treatment followed by three-day berzosertib treatment.
A. Drug screen plate layout and photographic images of the drug screen plates over time of regrowth after drug washout. **B.** and **C.** as (**A**).

Supplementary figure 7

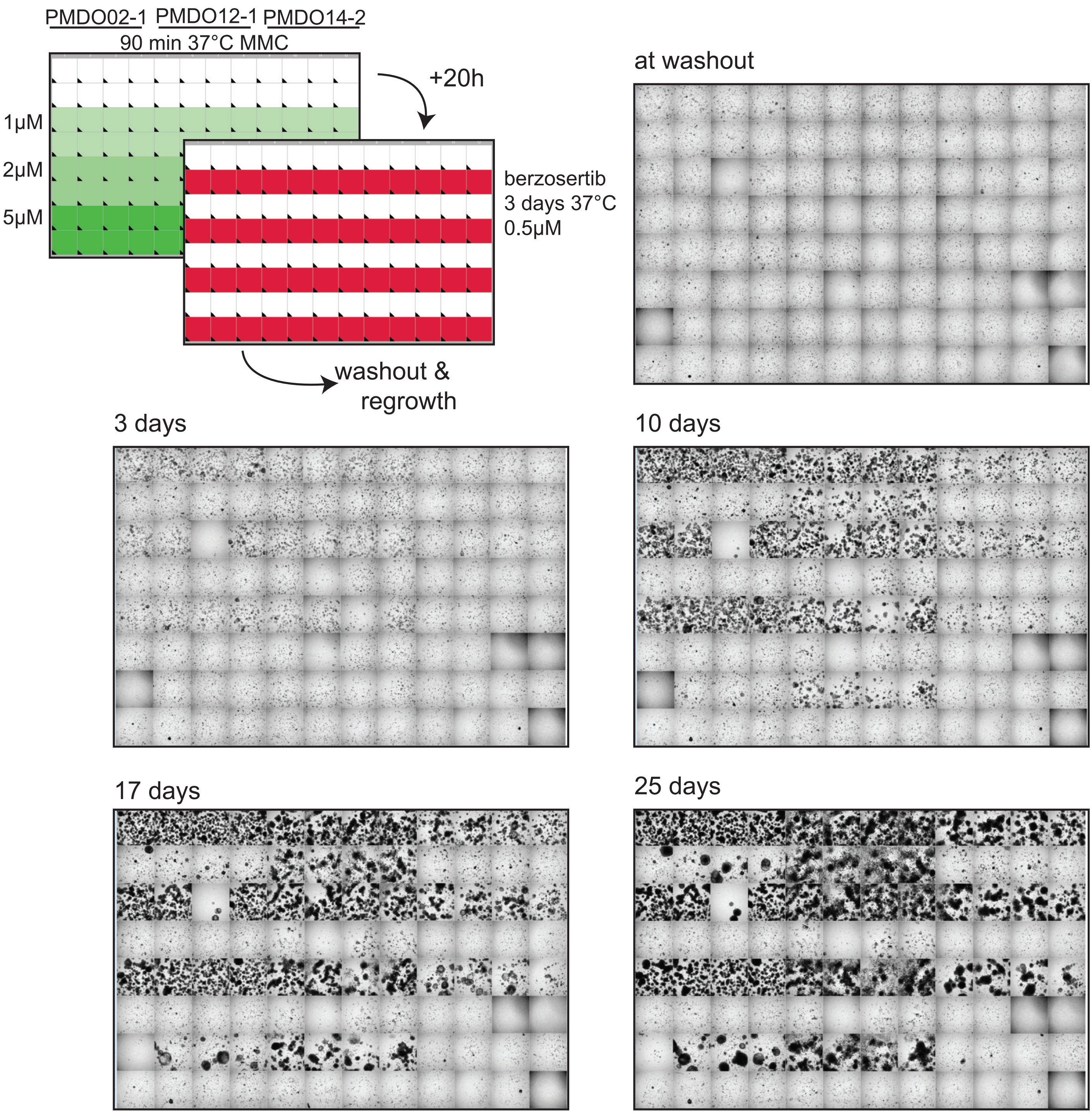

SF7. Outgrowth assays show synergistic effects of 90-minute 37°C MMC treatment followed by three-day berzosertib treatment.
Drug screen plate layout and photographic images of the drug screen plates over time of regrowth after drug washout.

Supplementary figure 8

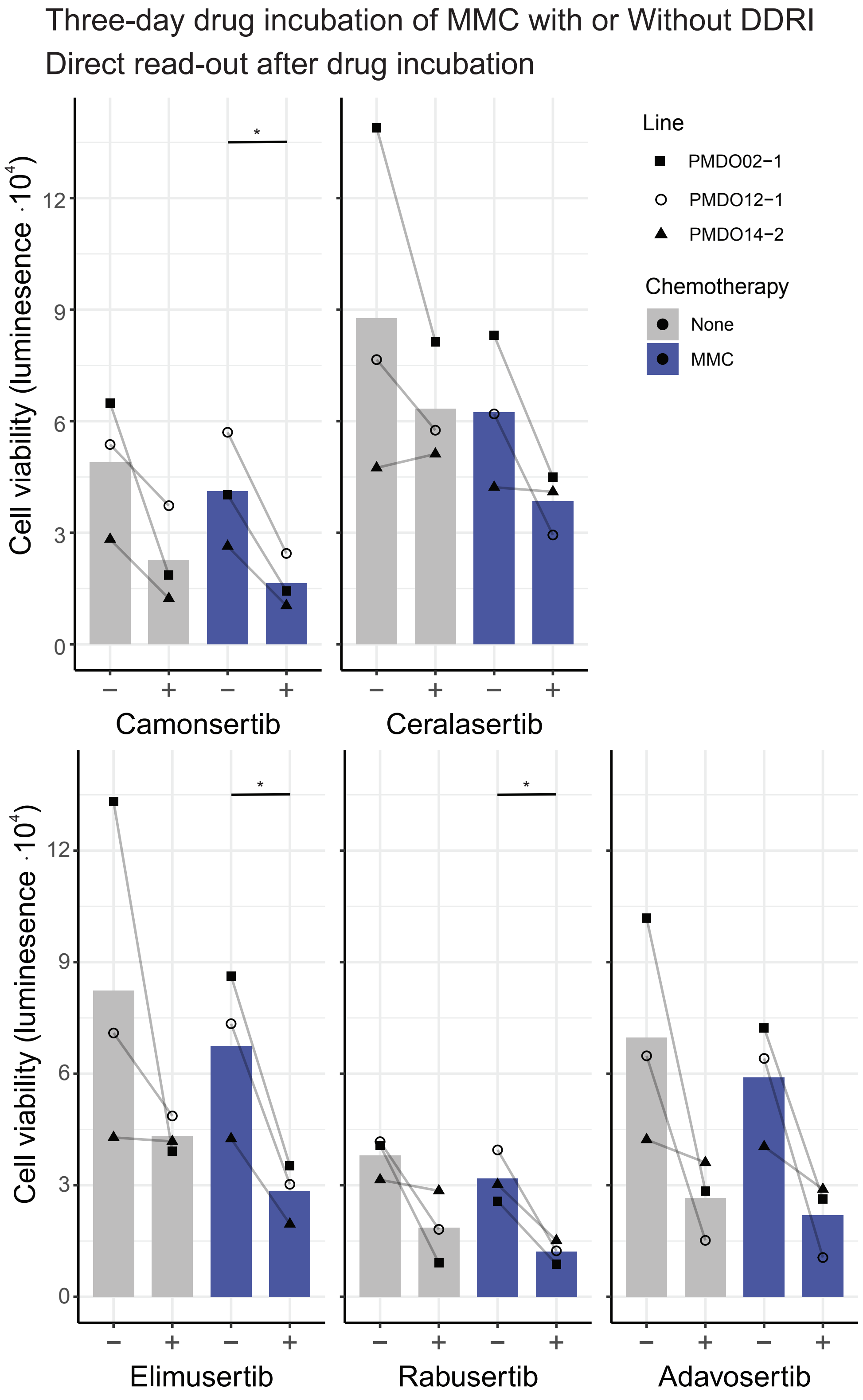

SF8. Three-day drug incubation with clinically relevant DDR inhibitors alone or in combination with MMC has limited effect on PMDO cell viability directly after treatment. CellTiter-Glo cell viability readout directly after 72 hour treatment with MMC (0.1μM), or DDR inhibitor camonsertib, ceralasertib, elimusertib, rabusertib, or adavosertib, alone or the combination, on three distinct PMDOs. Concentrations as in **ST5**. Graphs show the raw luminescence data. *P<0.05, one-way ANOVA with Tukey's and Dunnett's multiple comparisons.

Supplementary figure 9

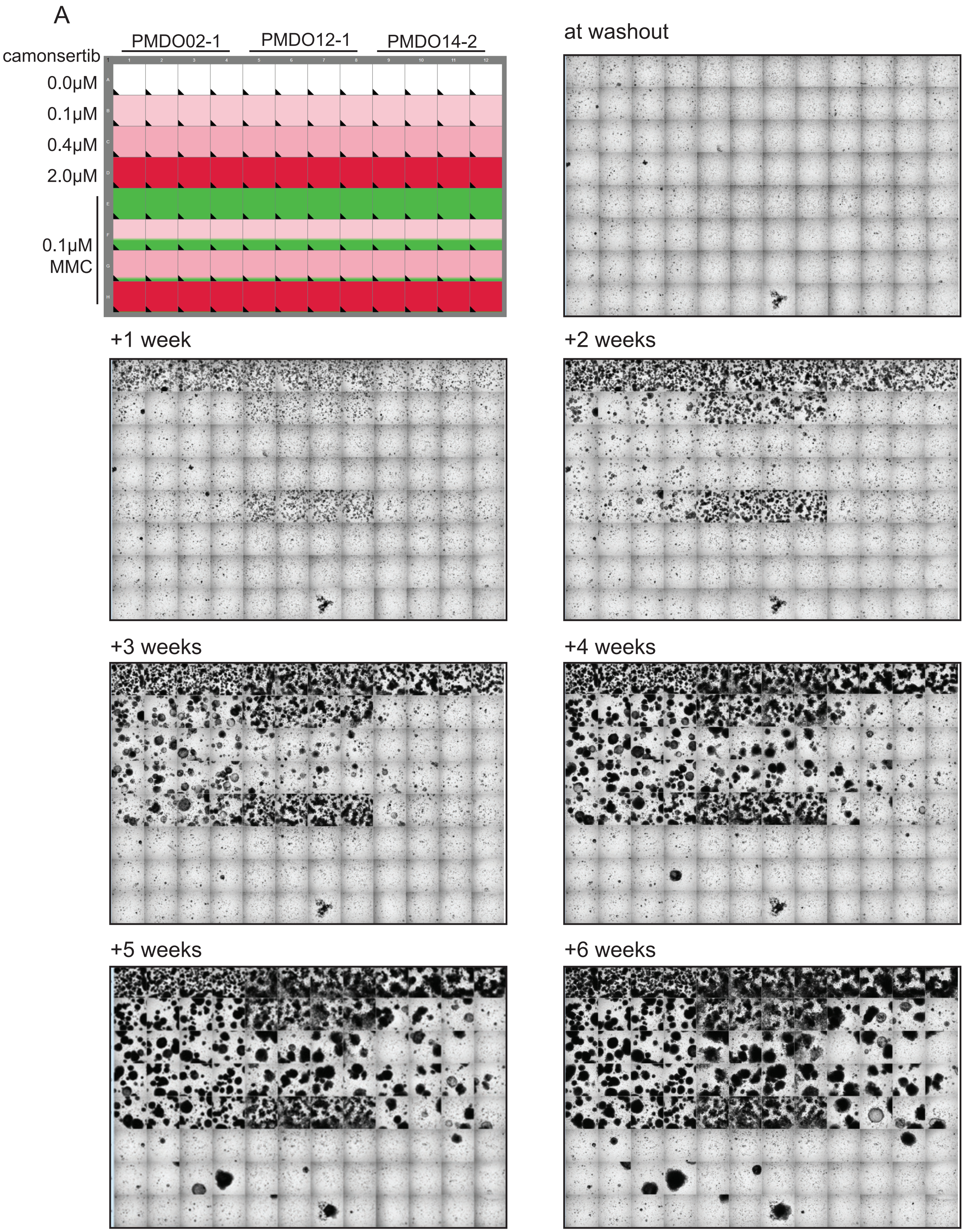

Supplementary figure 9

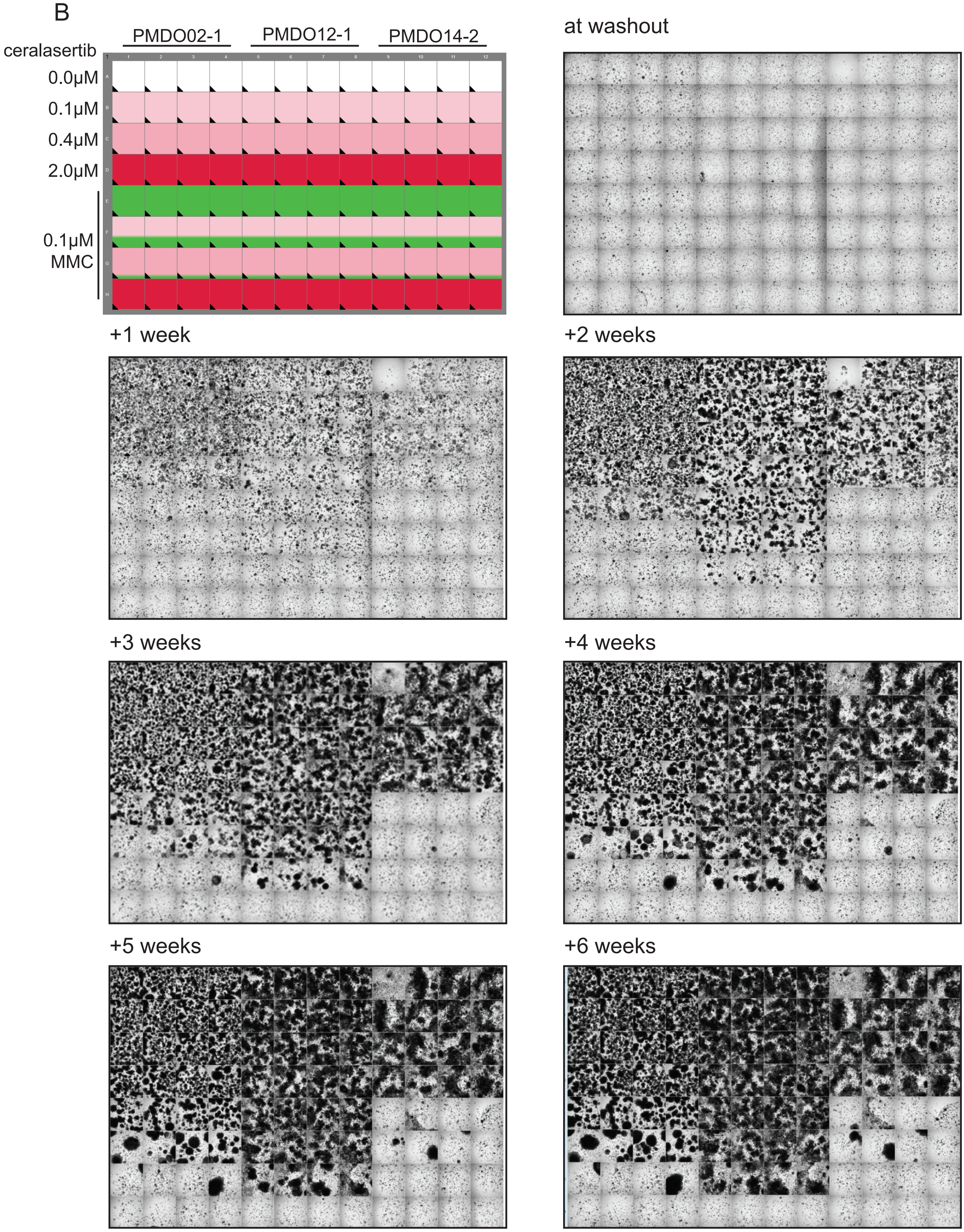

Supplementary figure 9

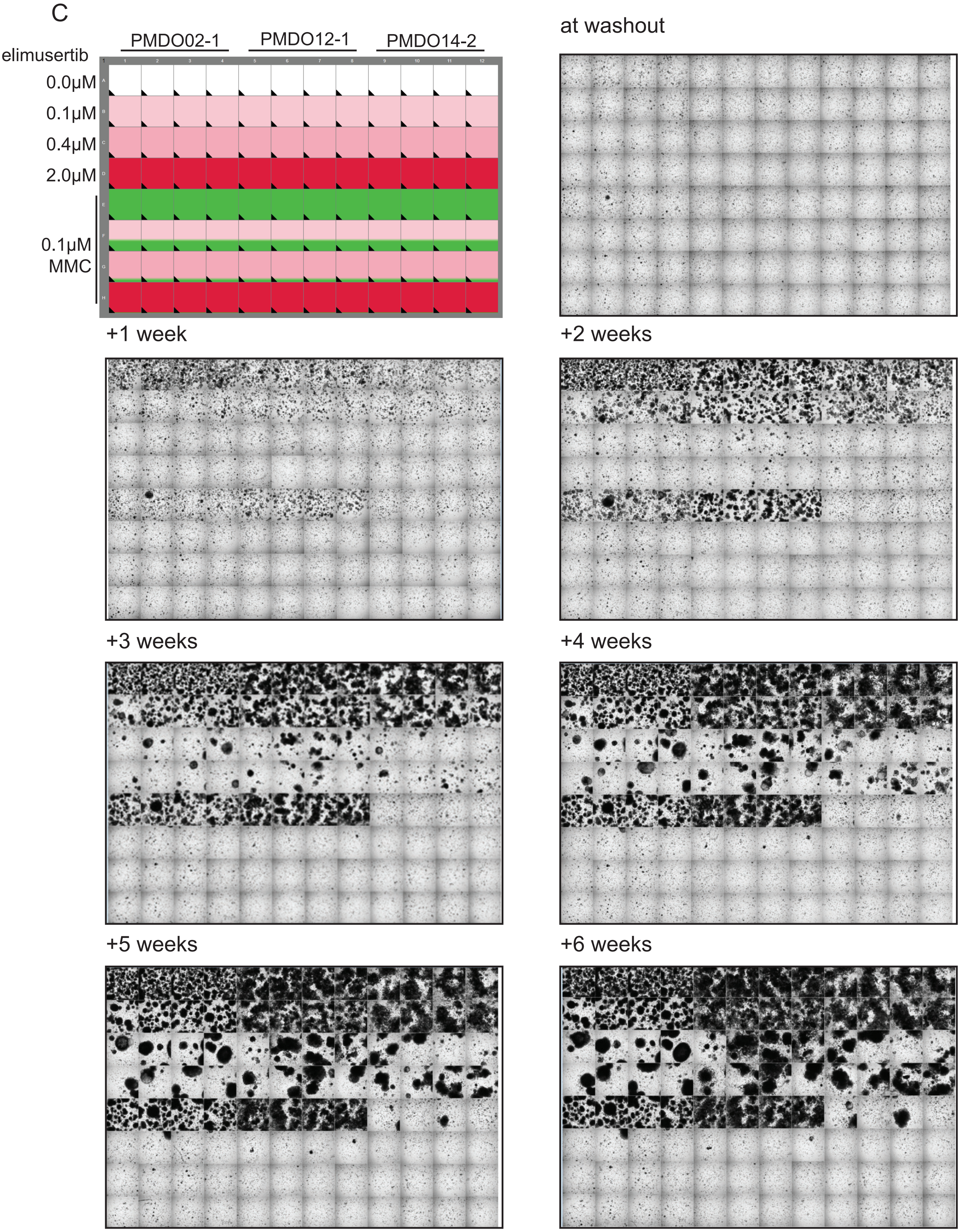

Supplementary figure 9

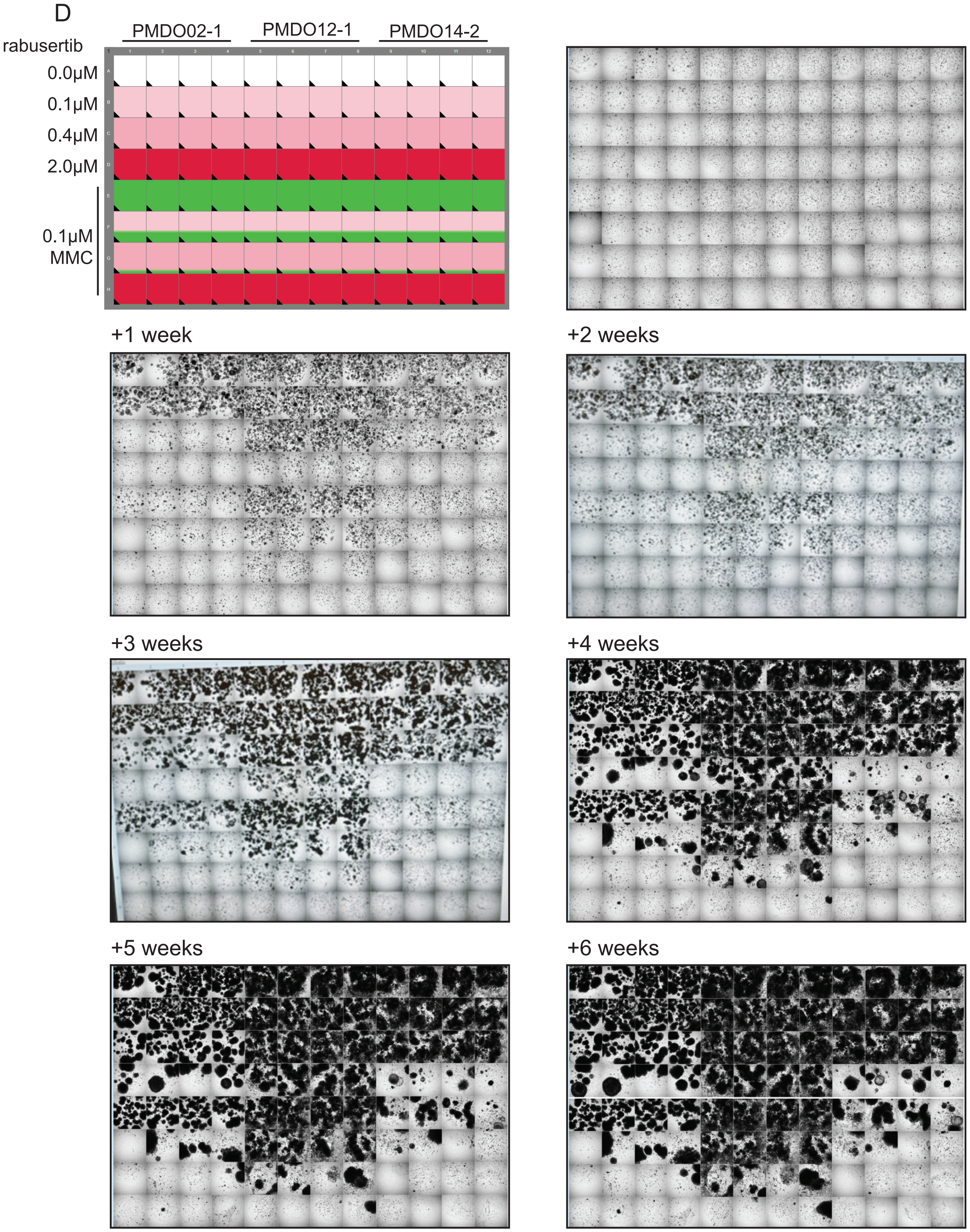

Supplementary figure 9

E

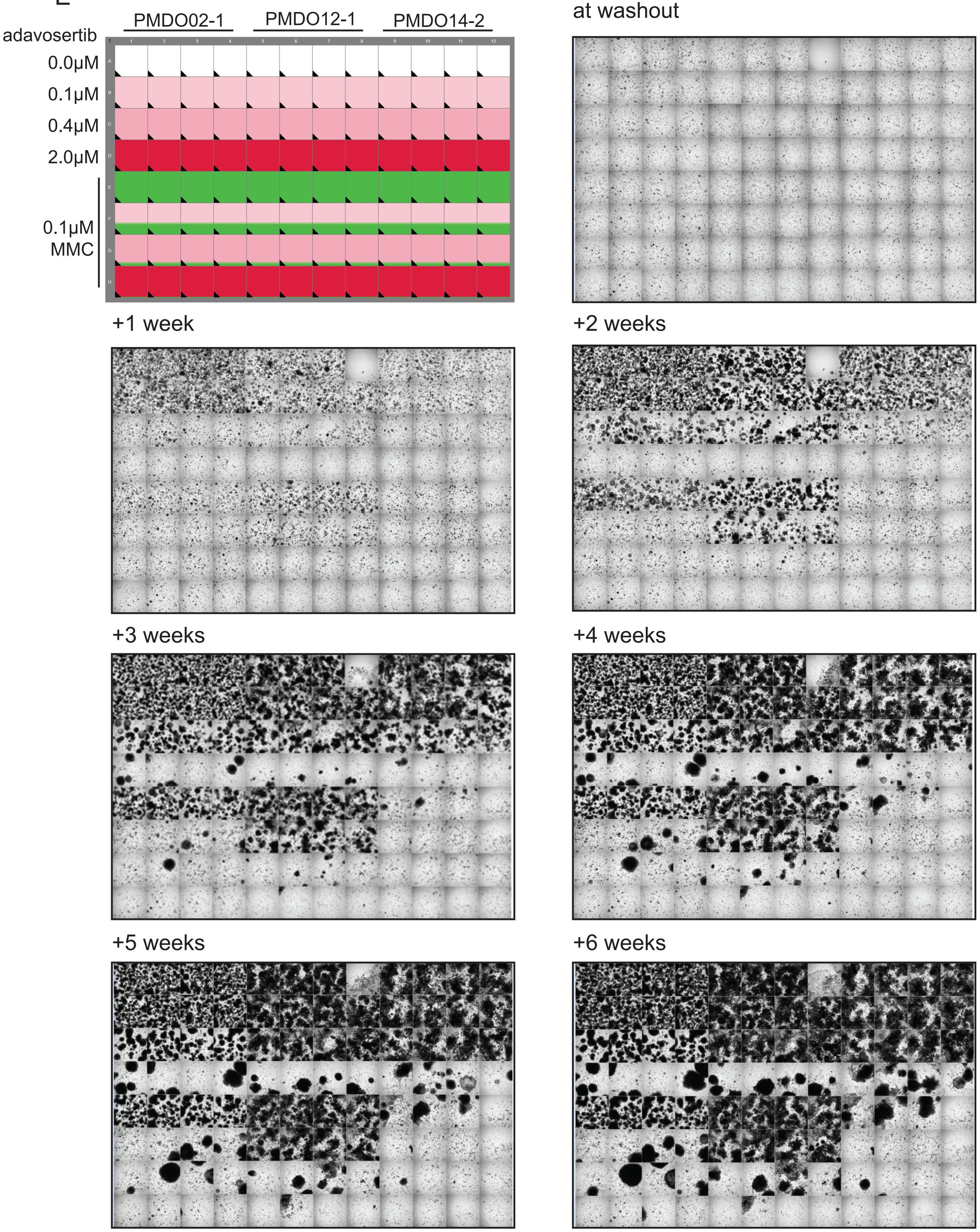

SF9. Regrowth assays show synergistic effects between MMC and DDRI after three-day drug treatment.

A-E. Drug screen plate layout and photographic images of the drug screen plates over time of regrowth after drug washout for camonsertib, caralasertib, elimusertib, rebusertib, and adavosertib, respectively.

Supplementary figure 10

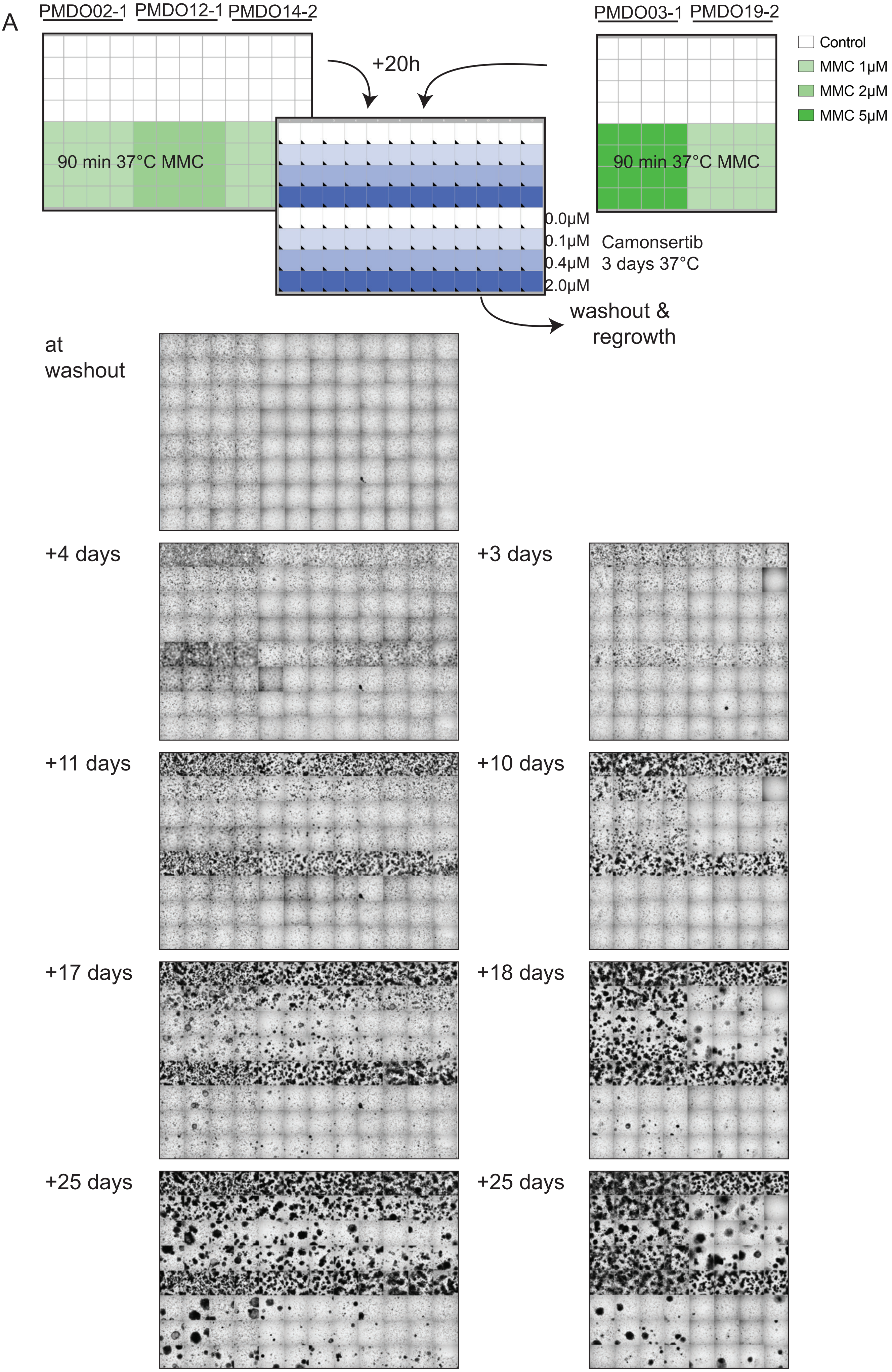

Supplementary figure 10

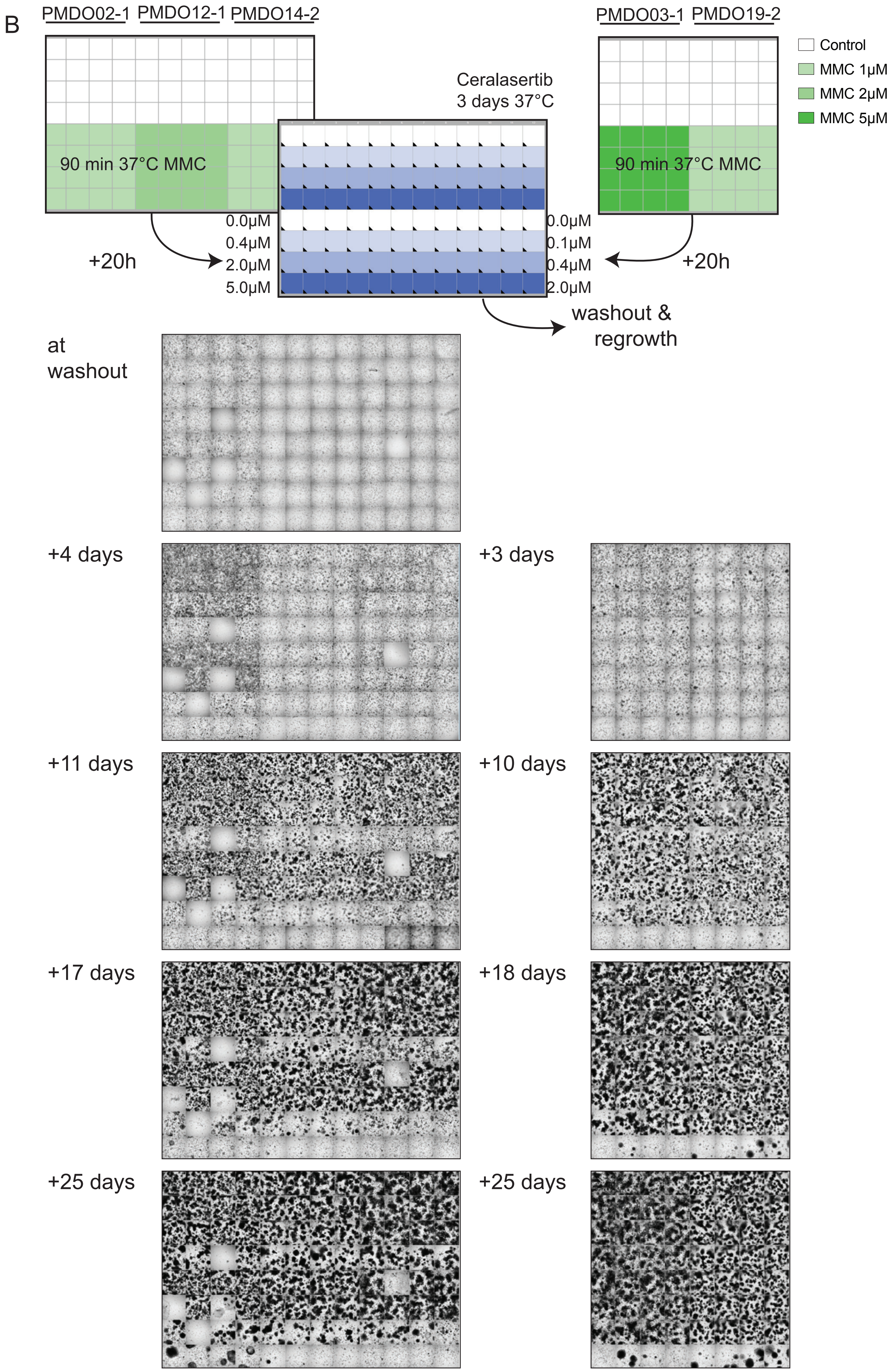

Supplementary figure 10

C

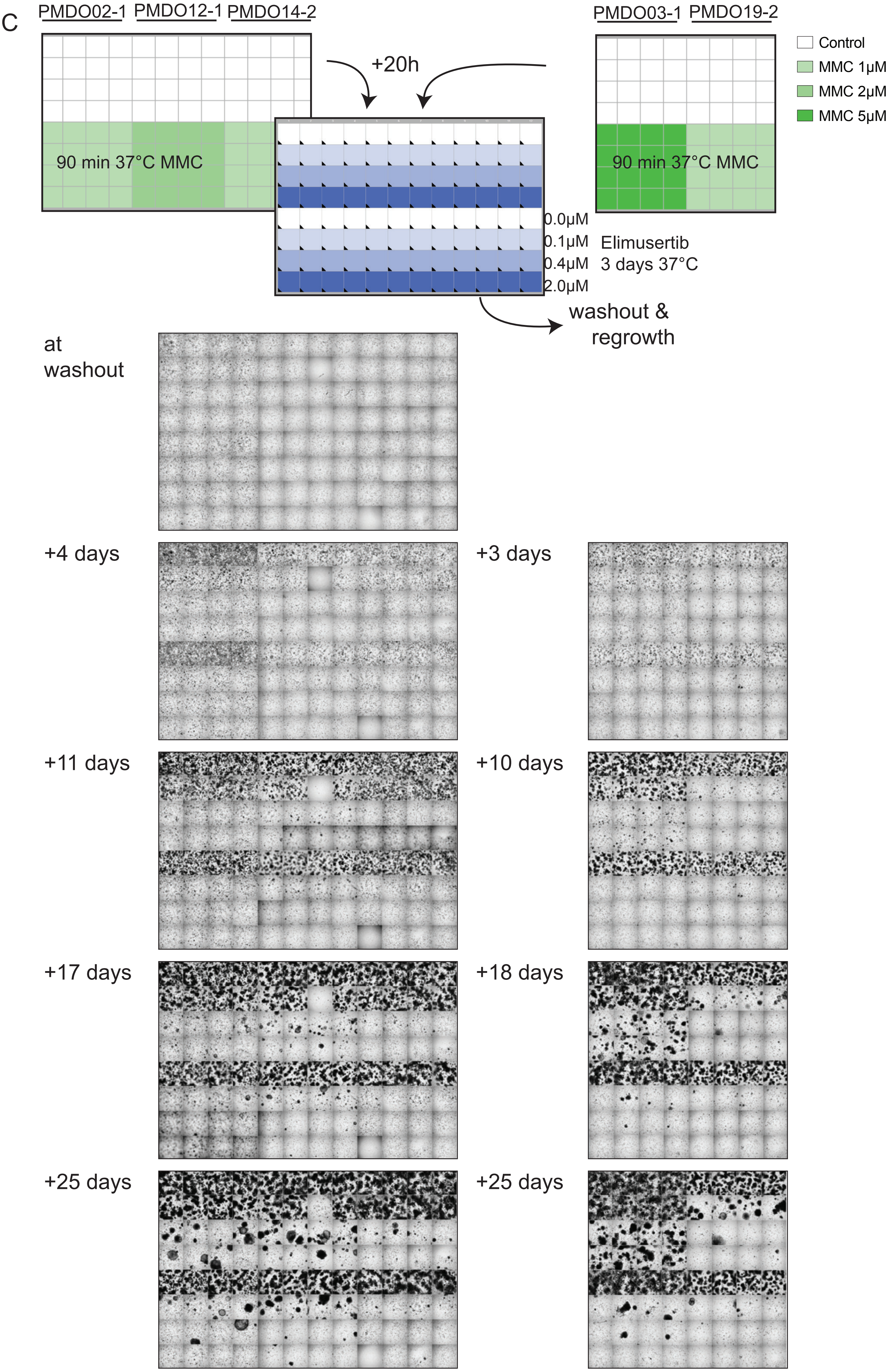

Supplementary figure 10

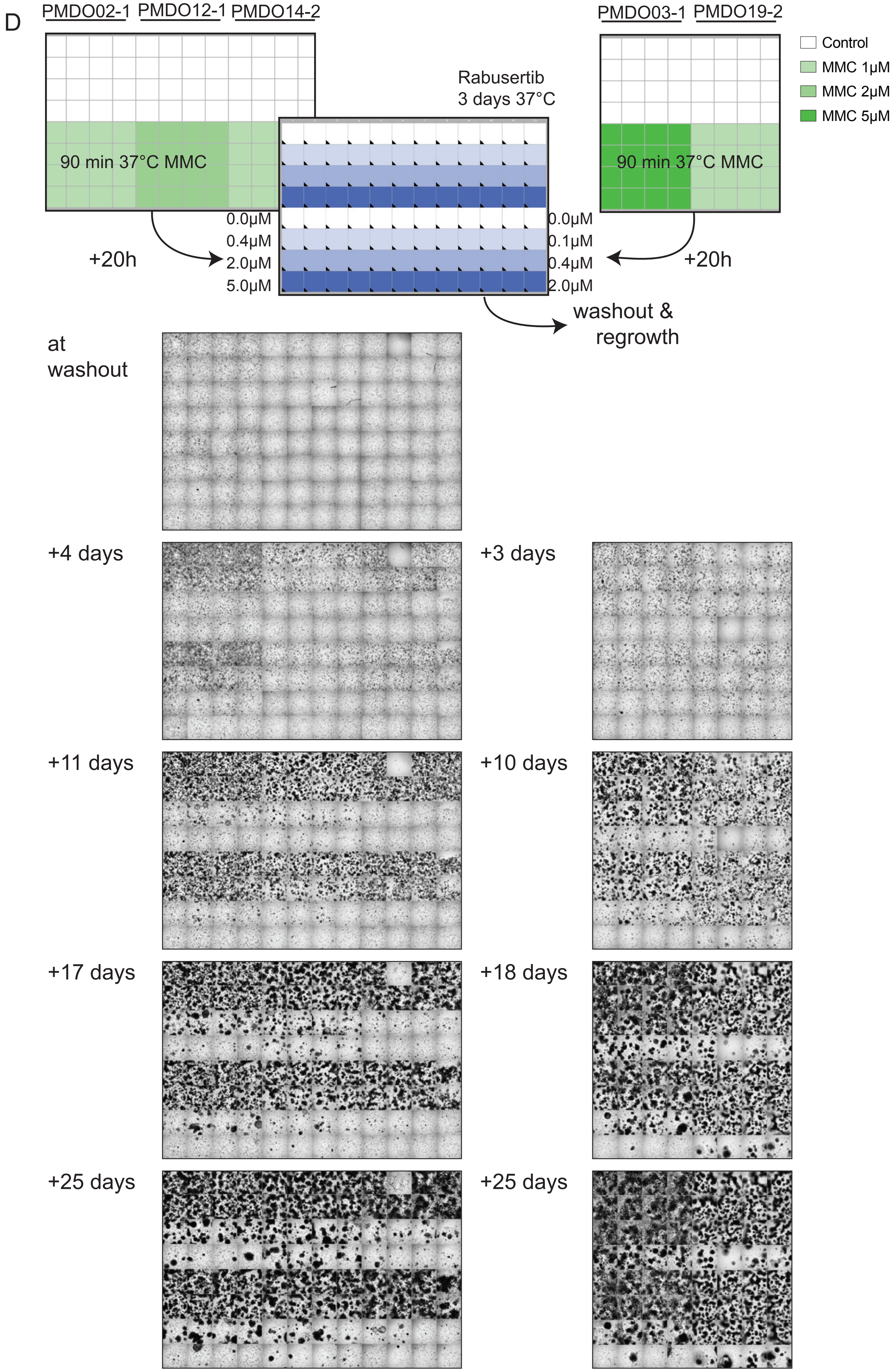

Supplementary figure 10

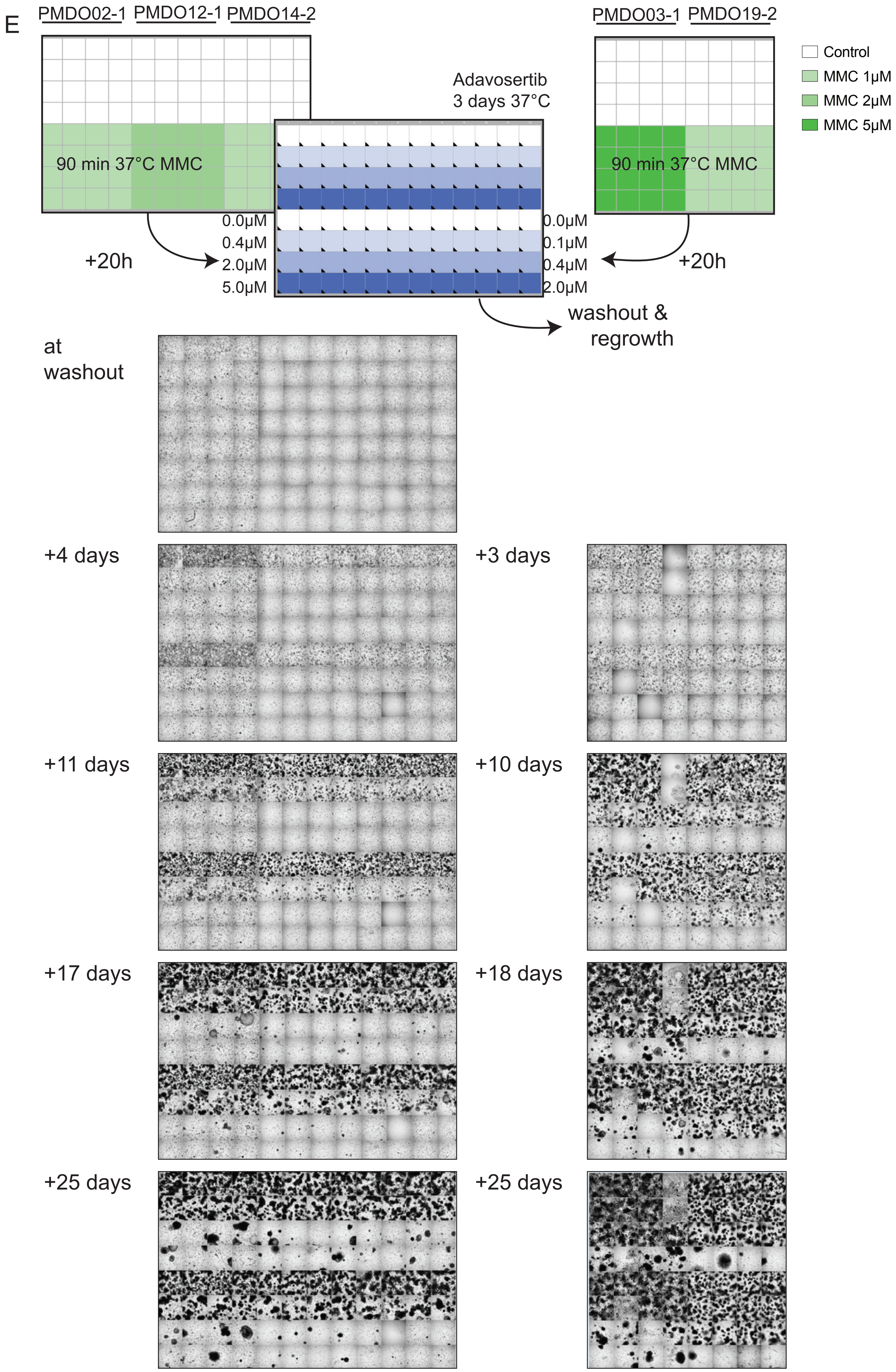

Supplementary figure 10

SF10. Outgrowth assays show synergistic effects of 90-minute 37°C MMC treatment followed by three-day DDR inhibitor treatment.

A-E. Drug screen plate layout and photographic images of the drug screen plates over time of regrowth after drug washout for camonsertib, caralasertib, elimusertib, rebusertib, and adavosertib, respectively.
